# Challenges and opportunities in clinical field research: A Bourdieusian analysis of Fiocruz’s experience in Brazil

**DOI:** 10.1371/journal.pone.0359217

**Published:** 2026-09-25

**Authors:** Renata Caldeira Diniz, Jocieli Malacarne, Cássia Ronise Senra Silva, Marcia Cristina Senra Marinho de Lima, Natalia Pacheco de Moraes, Thais Regina Araújo dos Santos, André Bastos Daher, Júlio Castro Alves de Lima e Silva, Karla Regina da Silva Gram

**Affiliations:** 1 Social Responsibility, Hospital Alemão Oswaldo Cruz, São Paulo, São Paulo, Brazil; 2 Clinical Research Platform, Vice-Presidency of Research and Biological Collections, Oswaldo Cruz Foundation (Fiocruz), Rio de Janeiro, Rio de Janeiro, Brazil; 3 Independent Scholar, Varginha, Minas Gerais, Brazil; UKM: Universiti Kebangsaan Malaysia, MALAYSIA

## Abstract

To identify and analyze the main challenges and opportunities of clinical field research from the perspective of Pierre Bourdieu’s reflexive sociology, using the experiences of the Fiocruz Clinical Research Platform in Brazil as a case study. A convergent mixed-methods study with a cross-sectional design was conducted involving 51 research team members linked to Fiocruz calls for proposals. Quantitative data were analyzed using descriptive statistics. Qualitative data underwent thematic analysis supported by MAXQDA, grounded in the analytical categories of field, *habitus*, and capital. The sample comprised predominantly women (76.5%) and highly qualified professionals with graduate degrees (80.4%). Logistical challenges were almost ubiquitous (96.1%), with access to remote locations being the most frequently cited barrier (82.4%), highlighting geographic isolation as a major constraint on knowledge production. Ethical challenges were reported by 52.9% of the agents, with obtaining the Informed Consent Form or Assent Form being the most frequent barrier (35.3%). This challenge was largely driven by participants’ comprehension difficulties (61.1%), misinformation (55.6%), and mistrust (50.0%). Notably, the study revealed an engagement paradox: although 90.2% of researchers perceived direct impact on the community as a significant opportunity, inclusive practices remained scarce. Specifically, 80.4% did not consult local leaders, and 88.2% did not integrate community residents into the field teams. The logistical and ethical barriers reflect structural vulnerabilities and capital asymmetries in the scientific field, where the researcher’s symbolic authority often legitimizes social distance from the territory. Consolidating clinical field research as a driver of equity within the Unified Health System (SUS) depends on the institutional capacity to convert scientific capital into social capital. It is imperative to transcend technocratic perspectives by institutionalizing operational risk management and strengthening symmetrical dialogues that recognize communities as subjects with legitimate cultural capital.

## Introduction

Clinical research in Brazil is a strategic pillar for the advancement of public health, a journey that began with pioneering investigations into infectious agents, such as the studies by Wucherer and Pirajá da Silva [1], and has progressively evolved into a robust ecosystem of technological innovation. Recently, this landscape was consolidated by Law No. 14,874/2024, which established the regulatory framework for research involving human subjects in the country. In Article 2, the aforementioned law defines clinical research as a systematic set of procedures designed to evaluate the safety and efficacy of technologies and care, as well as to assess the distribution of diseases and the impact of determinants on the health of the population [2]. This guideline represents the culmination of decades of regulatory development, guided by National Health Council (CNS) Resolutions 196/96 and 466/12, and by the work of the Brazilian Health Regulatory Agency (Anvisa) [3], which have brought the country into alignment with Good Clinical Practice (GCP).

In this context, the Oswaldo Cruz Foundation (Fiocruz) stands out as one of the leading health science and technology institutions in Latin America, with a 125-year history dedicated to scientific excellence. As a strategic arm of the Ministry of Health, the institution plays a fundamental role in technological sovereignty and the sustainability of the Unified Health System (SUS), operating in ten Brazilian states through 16 technical-scientific units focused on promoting health equity [4]. Given this extensive reach and expertise, Fiocruz’s institutional experience across a geographically heterogeneous territory provides valuable contextual insights that can inform broader discussions on clinical research governance in resource-limited settings, while acknowledging that direct transferability is inherently constrained by local systemic specificities.

Within the context of Fiocruz’s institutional activities, clinical field research stands out for its immersive approach, which encompasses the evaluation of technologies and new therapies, the distribution of diseases, and the analysis of the impact of health determinants in natural settings, such as communities, schools, and workplaces [5]. Unlike clinical trials, which are conducted primarily in controlled settings, this approach generates real-world evidence by considering the social and cultural dimensions that shape the epidemiological profile.

However, conducting these studies poses significant logistical challenges, requiring methodological adaptability in the face of infrastructure limitations in remote areas, especially in a country of continental dimensions such as Brazil [6]. Currently, Fiocruz’s organizational structure includes the Vice Presidency for Research and Biological Collections (VPPCB), which is responsible for proposing guidelines and promoting scientific research. Under its management, the Clinical Research Platform/Fiocruz acts as a strategic framework that ensures data quality and compliance with the fundamental ethical and regulatory requirements for technological development applicable to the SUS [7].

The Clinical Research Platform/Fiocruz actively participates in funding initiatives such as the Program for Excellence in Research (PROEP/PEC) and the INOVA Clinical Research Program, which fund interdisciplinary projects aimed at addressing priority public health diseases. It is based on the portfolio of projects approved through these strategic calls for proposals that this study defines its population, enabling a comprehensive assessment of the operation and obstacles of clinical field research in Brazil. These obstacles, while observed within specific Brazilian research settings, illustrate operational and logistical challenges that may resonate across developing health systems, where translating scientific evidence into public practice requires highly context-specific risk management.

Despite these obstacles, clinical field research offers unique opportunities to serve vulnerable populations, providing insights for the formulation of equitable public policies and promoting health surveillance that goes beyond the traditional biomedical model. For this scientific output to translate into concrete benefits for the management and formulation of public policies within the SUS, it is essential that decisions be grounded in solid evidence and the optimization of resources [8]. However, although such studies are scarce, it is necessary to systematically analyze the operational, ethical, and institutional challenges faced by field research teams. By addressing this need, this study provides a situated diagnosis of this institutional setting and offers an exploratory contribution to debates surrounding the operational sustainability of clinical research in resource-limited settings.

To understand the dynamics of this process, this study draws on Pierre Bourdieu’s conceptual triad: field, *habitus*, and capital. The choice of Bourdieusian sociology is justified because operational challenges, particularly in low- and middle-income countries, are rarely merely technical. They are direct reflections of asymmetries in power, prestige, and resource allocation—often invisible structures that underpin the scientific field and sociopolitical dynamics that purely biomedical or epidemiological frameworks frequently fail to capture. Therefore, Bourdieusian praxeology enables the articulation of the dialectical relationship between the structured structure (habitus) and the structuring structure (field). In other words, the approach seeks to analyze how objective structure and subjectivity are inherently relational and mutually reinforcing.

From this perspective, the field constitutes the objective dimension of social reality, taking the form of a structured space of positions whose properties are defined independently of the agents who occupy them at any given moment. The mediation between the objective structures of this field and the practical actions of the teams occurs through *habitus*, a system of durable and transferable dispositions that results from the internalization of the agents’ individual and professional trajectories. The *habitus* functions as a matrix that organizes perceptions and practices, ensuring that responses to unforeseen challenges in the research field are orchestrated and adapted, even without conscious adherence to rigid rules. The teams’ capacity for maneuver, however, is mediated by capital. Cultural capital, embodied in academic credentials and technical expertise, and social capital, derived from collaborative networks, constitute resources that, when legitimized by peers, are transformed into symbolic capital [9,10], conferring the authority to speak and the prestige necessary for the successful conduct of research.

Thus, the central objective of this study is to identify and analyze the main challenges and opportunities of clinical field research projects from a Bourdieusian perspective, using the experiences of the Fiocruz Clinical Research Platform as a case study. This assessment seeks to systematize lessons learned that will foster procedural adaptability and improve the management of scientific projects, providing essential insights for decision-making in the realm of public health and the strengthening of the SUS.

## Materials and methods

### Ethics statement

The study was approved by the Research Ethics Committee of the Oswaldo Cruz Institute (CAAE: 89038525.6.0000.5248; Opinion No. 7.698.450) (S1 Appendix). All participants provided the Informed Consent Form (ICF) digitally.

### Study design

This study adopted a convergent mixed-methods design with a cross-sectional approach, characterized by the simultaneous collection of quantitative and qualitative data through a single instrument. This approach allowed for conducting the performance of independent analyses that were integrated during the interpretation phase to provide a multidimensional understanding of the object of study. The analysis was based on Pierre Bourdieu’s reflexive sociology, using the analytical categories of field, habitus, and capital to reveal the structural dynamics, subjective dispositions, and power relations that organize clinical field research at Fiocruz.

### Study population and sample

The study population consisted of researchers and members of research teams —regardless of their role or position—associated with six field clinical research projects funded by Fiocruz calls for proposals: the Program for Excellence in Research/Clinical Research and Trials (PROEP/PEC 2020) and the INOVA calls for Schistosomiasis (No. 9/2022) and Chagas Disease (No. 5/2023).

Recruitment was conducted electronically via invitations sent by email and WhatsApp, identified as the primary communication channels of the surveyed teams. The invitation detailed the study’s objectives, the voluntary nature of participation, confidentiality guarantees, and the risks and benefits involved, providing a link to the ICF. In strict compliance with the ethical principles of research involving human subjects, consent was obtained in digital format; access to the questionnaire was contingent upon confirmation of electronic acceptance, ensuring the autonomy of the participants and full compliance with current regulatory standards.

The sample, consisting of 51 participants, was selected using non-probability and convenience sampling, comprising agents over the age of 18 who were involved in at least one of the identified clinical research projects. Participants with less than one year of field experience were excluded, a measure necessary to ensure that individuals possessed the minimum experience required to incorporate the specific habitus of this social microcosm. From a praxiological perspective, this maturity in the career trajectory is essential for the agents’ perceptions to reflect the enduring dispositions and the schemes of perception and action that constitute the field.

The selection of these professionals was based on their strategic position within the scientific field, conceived as a space of objective relationships and competitive struggles for control over specific forms of capital—particularly symbolic capital, which confers authority and legitimate recognition among peers.

Although the sample size (*n* = 51) may limit the statistical power for universal generalizations, the study design prioritized the representativeness of the participants within Fiocruz’s specific scientific field. Due to the relatively small sample size, the findings are largely considered hypothesis-generating, establishing a baseline for broader future investigations. From the perspective of reflexive sociology, this approach allowed for a situated and in-depth analysis of capital asymmetries and the structural barriers faced. Thus, the statistical data provided a basis for revealing how the unequal distribution of these resources shapes power strategies and relations within the field.

### Data collection and instrument

Data were collected using a semi-structured electronic questionnaire (S2 Appendix) hosted on the Research Electronic Data Capture (REDCap) platform, on a Fiocruz institutional server. The use of this tool was based on its ability to provide an intuitive interface for validated data capture, audit trails for tracking all procedures, automated export to statistical software packages, and protocols for secure data integration [11]. Recruitment took place via individual invitations sent by email and instant messaging apps. The instrument was organized into five thematic domains: (1) sociodemographic and professional profile; (2) field research challenges; (3) ethics, quality, and safety; (4) opportunities; and (5) conceptual perceptions. The complete structure of the variables, including their attributes and branching logic, is detailed in the REDCap Data Dictionary Codebook, available as Supplementary Material (S3 Appendix).

Content validity was established through review by two expert researchers, followed by a pilot study to refine the logical flow and ensure alignment with the respondents’ professional habitus. The closed-ended questions used dichotomous and multiple-choice variables to measure the prevalence of barriers. Meanwhile, open-ended fields labeled “other” were strategically designed to capture information that might not have been covered by the closed categories, offering agents a space to record unusual, unexpected, or unspoken elements of their daily practice.

### Data analysis

The study employed a mixed-methods approach based on analytical complementarity, seeking to overcome the limited perspective of a single method to capture the complexity of social relationships in the scientific field. Quantitative data were analyzed using descriptive statistics (absolute and relative frequencies) in the Jamovi software. Exclusively for the analysis of qualitative data derived from open-ended responses and optional “other” fields, completed by 13 participants, the MAXQDA 26 software was employed, following a hierarchical thematic coding protocol that linked deductive categories from the theoretical framework with inductive themes emerging from the agents’ practice (S1 Fig). To ensure methodological rigor and analytical consistency, coding was conducted independently by two of the authors and subjected to peer validation to resolve any discrepancies through consensus. The anonymized raw dataset, as well as the details of the qualitative analysis categories, are available in the Supplementary Information (S1 Dataset and S1 Appendix).

The coding framework organized the information into two analytical axes. The first, focused on mitigating challenges, concentrated on mapping proactive strategies interpreted as manifestations of practical sense and scientific habitus, revealing how agents mobilize their cultural and technical capital to ensure ethical integrity and quality standards. The second axis was dedicated to the dynamics of challenges and opportunities, performing a multidimensional categorization of political, psychosocial, and operational barriers understood as reflections of power relations and structural constraints within the field, contrasting them with the potential for innovation and the tensions between strategies of conservation and subversion of the established order.

The final analysis compared quantitative prevalence data with qualitative perceptions, enabling a reflexive sociological examination of how one’s position within the field and the accumulation of capital influence how challenges in clinical research within that field are addressed.

## Results

### Participant profile

Between July and August 2025, 67 participants were recruited, of whom 51 comprised the final sample. The 16 exclusions comprised: participants who signed the ICF but did not complete the questionnaire (n = 7), duplicate questionnaire entries (n = 3), and failure to meet the eligibility criterion of having at least one year of clinical field research experience (n = 6). As shown in Table 1, the sample was predominantly female (76.5%, *n* = 39), with a mean age of 40 years and a highly qualified professional background, with 80.4% (*n* = 41) holding a completed graduate degree. Regarding self-reported ethnic and racial background, 68.6% (*n* = 35) identified as white, 25.5% (*n* = 13) as mixed-race (Parda), and 3.9% (*n* = 2) as Black.

**Table 1:** Sociodemographic and professional characteristics of research participants (*n* = 51).

Variable	*n* (%)
**Age (years)**	
22–34	15 (29.4)
35–47	21 (41.2)
48–60	9 (17.6)
61–73	6 (11.8)
**Sex**	
Female	39 (76.5)
Male	12 (23.5)
**Race/Color (self-reported)**	
White	35 (68.6)
Mixed-race (Parda)	13 (25.5)
Black	2 (3.9)
Not reported	1 (2.0)
**Education Level**	
Completed Graduate Studies	41 (80.4)
Incomplete Graduate Studies	3 (5.9)
Completed Undergraduate Degree	5 (9.8)
Incomplete Undergraduate Degree	1 (2.0)
High School	1 (2.0)
**Academic Background**	
Biological Sciences	17 (33.3)
Pharmacy	10 (19.6)
Biomedicine	8 (15.7)
Nursing	6 (11.8)
Other backgrounds^*a*^	10 (19.6)
**Professional Experience (years)** ^***b***^	
1–4	23 (46.9)
5–9	13 (26.5)
10–14	5 (10.2)
15–24	2 (4.1)
25–29	2 (4.1)
> 30	4 (8.2)
**Geographic Region (Brazil)**	
Southeast	28 (54.9)
Northeast	18 (35.3)
South	3 (5.9)
North	2 (3.9)
Central-West	0 (0.0)

Notes: Percentages may not total 100% due to rounding.

a Other backgrounds include: Veterinary Medicine, Engineering, Physiotherapy, Nutrition, Medicine, and Technical Training.

b Missing data: Two participants did not provide information on professional experience (n = 49 for this category).

The most common fields of study were Biological Sciences (33.3%, *n* = 17), Pharmacy (19.6%, *n* = 10), and Biomedicine (15.6%, *n* = 8). In terms of experience, 46.9% (*n* = 23) had between 1 and 4 years of experience, while only 8.2% (*n* = 4) had more than 30 years of experience. Geographically, there was a concentration in the Southeast region (54.9%, *n* = 28), with low representation from the North and Midwest regions.

### Challenges in clinical field research

**Fig 1:**
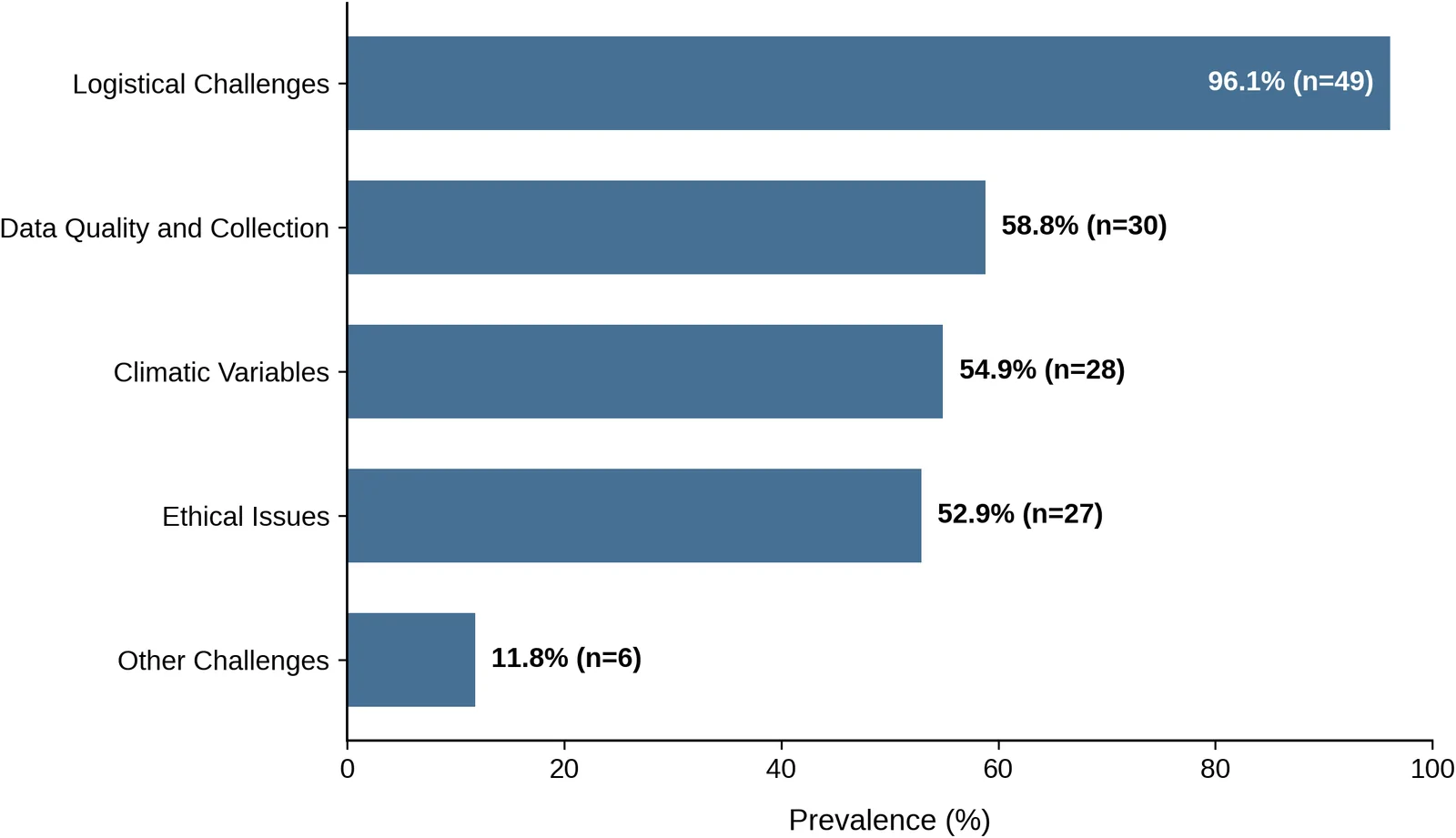
Prevalence of challenges encountered by researchers in conducting clinical field research (*n* = 51).

Nearly all participants (96.1%, *n* = 49) reported encountering logistical challenges during the study, followed by challenges related to data quality and collection (58.8%, *n* = 30), climatic variables (54.9%, *n* = 28), ethical issues (52.9%, *n* = 27), and other challenges (11.8%, *n* = 6) (Fig 1).

Regarding logistical challenges, access to remote locations was the most frequently cited category, mentioned by 42 participants (82.4%), highlighting geographic isolation as the main factor in logistical difficulties in this context. This was followed by time constraints and costs, each mentioned by 29 participants (56.9%). Transport of biological samples (n = 27, 52.9%), ineffective communication (n = 23, 45.1%), and equipment contingencies (n = 22, 43.1%) were also quite prevalent barriers. Notably, a small subgroup of participants (n = 2, 3.9%) reported not having faced any logistical challenges. The complete typology and distribution of these specific logistical barriers are detailed in Table 2.

**Table 2:** Typology and distribution of specific logistic challenges in clinical field research (n = 51).

Types of logistic challenges	Frequency of occurrence n (%)
Access to remote locations	42 (82.4%)
Limited time	29 (56.9%)
Costs	29 (56.9%)
Sample transportation	27 (52.9%)
Ineffective communication	23 (45.1%)
Transportation	23 (45.1%)
Equipment contingencies	22 (43.1%)
Team meals	21 (41.2%)
Team accommodation	19 (37.3%)
Document storage	18 (35.3%)
Difficulty in obtaining authorizations	17 (33.3%)
Lack of health infrastructure	17 (33.3%)
Security and risks	15 (29.4%)
Supply management	4 (7.8%)
Electricity	3 (5.9%)

Notes: Data are presented as n (%), representing the participants who answered “Yes” to experiencing the specific challenge. Percentages were calculated based on the total sample size (n = 51).

A sub-group of 2 participants (3.9%) reported experiencing no logistic challenges across any of the categories listed; therefore, they are not represented in the individual frequencies above.

### Prevalence and types of ethical challenges

Overall, 52.9% (*n* = 27) of participants reported experiencing at least one ethical challenge, while 47.1% (*n* = 24) did not report such challenges. The distribution of specific ethical challenges is summarized in Table 3.

**Table 3:** Distribution of specific ethical challenges reported by participants (n = 51).

Types of ethical challenges	Frequency of occurrence n (%)
Social, cultural and value differences	16 (31.4%)
Ensuring anonymity and confidentiality	4 (7.8%)
Impact on the local community	10 (19.6%)
Obtaining the ICF / AF	18 (35.3%)
Institutional pressures or conflicts of interest	3 (5.9%)
Power relations	2 (3.9%)
Data use and sharing	8 (15.7%)

Notes:

Data are presented as n (%), representing the participants who answered “Yes” to experiencing the specific challenge.

ICF/AF: Informed Consent Form / Assent Form.

A sub-group of 24 participants (47.1%) reported experiencing no ethical challenges across any of the categories listed; therefore, they are not represented in the individual frequencies above.

Percentages were calculated based on the total sample size (n = 51).

Among the specific types of ethical challenges identified, the most common was the process of obtaining the Informed Consent Form (ICF) or Assent Form (AF)—the latter applies to minors under 18 or individuals with impaired judgment—reported by 18 participants (35.3%). This was followed by social, cultural, and value differences (*n* = 16, 31.4%), the impact on the local community (*n* = 10, 19.6%), and issues regarding data use and sharing (*n* = 8, 15.7%). In contrast, other challenges were reported less frequently within the sample, including ensuring anonymity and confidentiality (*n* = 4, 7.8%), institutional pressures or conflicts of interest (*n* = 3, 5.9%), and power relations (*n* = 2, 3.9%).

### Barriers to the informed consent process

Out of the 51 participants, 18 (35.3%) reported difficulties in obtaining the Informed Consent Form or Assent Form (ICF/AF). When investigating the specific reasons within this subgroup (*n* = 18), the most prevalent barrier was participants’ difficulty in understanding the study information (61.1%, *n* = 11), followed closely by misinformation (55.6%, *n* = 10) and mistrust of the research or researchers (50.0%, *n* = 9). Furthermore, obstacles in clarifying research objectives and methods (33.3%, *n* = 6) and language barriers (22.2%, *n* = 4) were also identified as relevant contributors to these challenges, as detailed in Table 4. These results suggest that such communication and relationship obstacles act as constraints on the autonomy of participants and the effectiveness of ethical dialogue in the field.

**Table 4:** Distribution of specific barriers to the informed consent process among participants who reported difficulties (n = 18).

Types of specific barriers	Frequency n (%)
Difficulty of understanding	11 (61.1%)
Misinformation	10 (55.6%)
Mistrust	9 (50.0%)
Clarifying research objectives and methods	6 (33.3%)
Language barriers	4 (22.2%)

Notes:

Data are presented as n (%).

Percentages were calculated based on the sub-group of participants who reported experiencing difficulties in obtaining the ICF / AF (n = 18).

ICF/AF: Informed Consent Form / Assent Form.

### Qualitative analysis of field research challenges

The qualitative analysis of the optional open-ended responses (the “other” fields, *n* = 13), processed using MAXQDA 26 software, enabled the identification of dimensions that transcend the closed categories of the questionnaire, revealing the logic of practices and the tensions inherent in the investigated field. The findings were structured into the analytical axes established in the methodology, reflecting the dialectic between the structural barriers of the scientific field and the agents’ dispositions (*habitus*).

Within the logistical and operational scope, specific challenges such as the adaptation of data collection instruments (*n* = 1), risks of data loss and synchronization failures (*n* = 1), transportation of essential documents (*n* = 1), and difficulties in sample collection among specific populations (*n* = 1) operate as direct barriers to knowledge production. Such obstacles reflect disparities in geographic capital and the precariousness of national infrastructure in the face of the demands of scientific rigor.

These dynamics are permeated by the field of power, where the scarcity of human capital manifests as inexperienced teams (*n* = 2) and a lack of qualified personnel (*n* = 2). The reliance on support from local governance (*n* = 2) and the difficulties of working with vulnerable populations (*n* = 2) demonstrate the constant need for negotiation of social capital. Additional factors, such as political pressures, precarious health infrastructure, occupational risks, and the psychological impact on the team (*n* = 1 each), expose the struggles for institutional legitimacy within the territory.

In the ethical domain, interactions revealed significant symbolic distancing, highlighted by signature fatigue and bureaucracy (*n* = 2), as well as challenges in protocol adherence (*n* = 2). Reports of information asymmetry and mistrust (*n* = 1) and obstacles in the re-consent process (*n* = 1) suggest that the researcher’s accumulation of cultural capital can generate symbolic violence when bureaucratic rituals and specialized language restrict participants’ autonomy.

### Perception of opportunities in clinical field research

**Fig 2:**
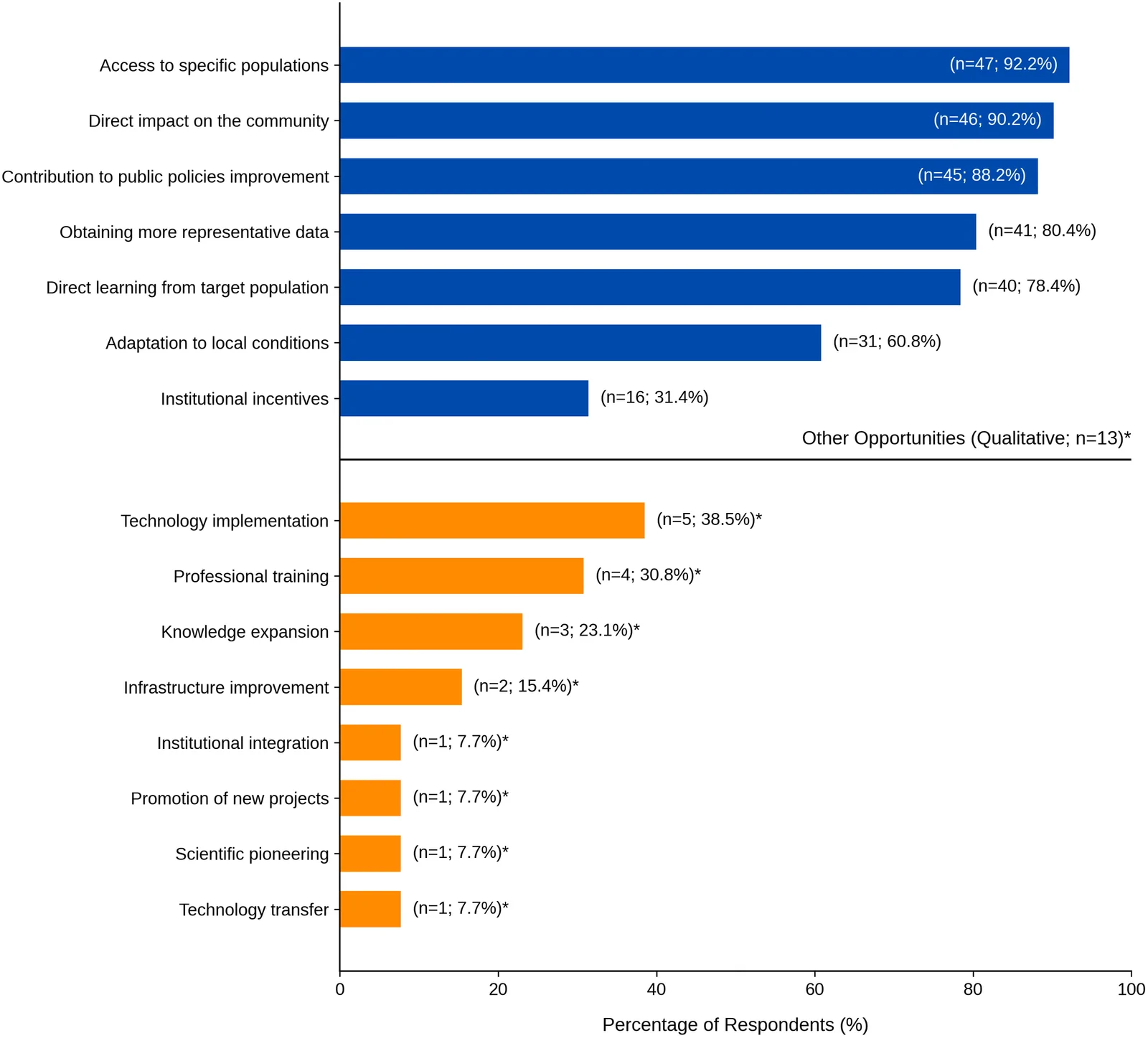
Distribution of identified opportunities and benefits reported by participants. Blue bars indicate predefined quantitative categories based on the total study sample (*n* = 51). Orange bars indicate emerging qualitative categories (“Other Opportunities”), calculated exclusively from the subgroup that provided open-ended responses (n = 13), denoted by an asterisk (*). Values represent absolute frequency (n) and percentage (%).

The analysis revealed a high prevalence of perceived opportunities among participants (*n* = 51) regarding the conduct of clinical field research (Fig 2). The most significant opportunities identified were access to specific populations (*n* = 47; 92.2%), direct impact on the community (*n* = 46; 90.2%), and contribution to the improvement of public policies (*n* = 45; 88.2%). Additionally, there was a high frequency in valuing the acquisition of more representative data (80.4%) and direct learning from the target population (78.4%). Although adaptation to local conditions was recognized by 60.8% of the respondents, institutional incentives emerged as the least cited opportunity among the predefined categories (31.4%).

Additionally, the qualitative analysis of open-ended responses (*n* = 13) conducted via MAXQDA software allowed the identification of specialized opportunities. These findings suggest that field research operates as a mechanism for converting technical competencies into symbolic and scientific capital, particularly through the implementation of technology (38.5%, *n* = 5), professional training (30.8%, *n* = 4), expansion of knowledge (23.1%, *n* = 3), and infrastructure improvement (15.4%, *n* = 2). Additional references to pioneering initiatives, institutional integration, and technology transfer (7.7%, *n* = 1 each) reinforce that immersive fieldwork consolidates the institution’s symbolic authority within the healthcare system.

Faced with the vulnerabilities of the field, researchers mobilize their scientific *habitus* through “practical sense”, prioritizing knowledge translation and feedback to the community (*n* = 2) as central acting strategies. This movement is complemented by practices of technical adaptation, integration with local management, and strict observance of Good Clinical Practice (*n* = 1 each). These actions reflect a proactive effort to convert accumulated cultural capital into operational viability and ethical integrity when confronted with the vicissitudes of the field.

### Adoption of inclusion practices and community trust

**Fig 3:**
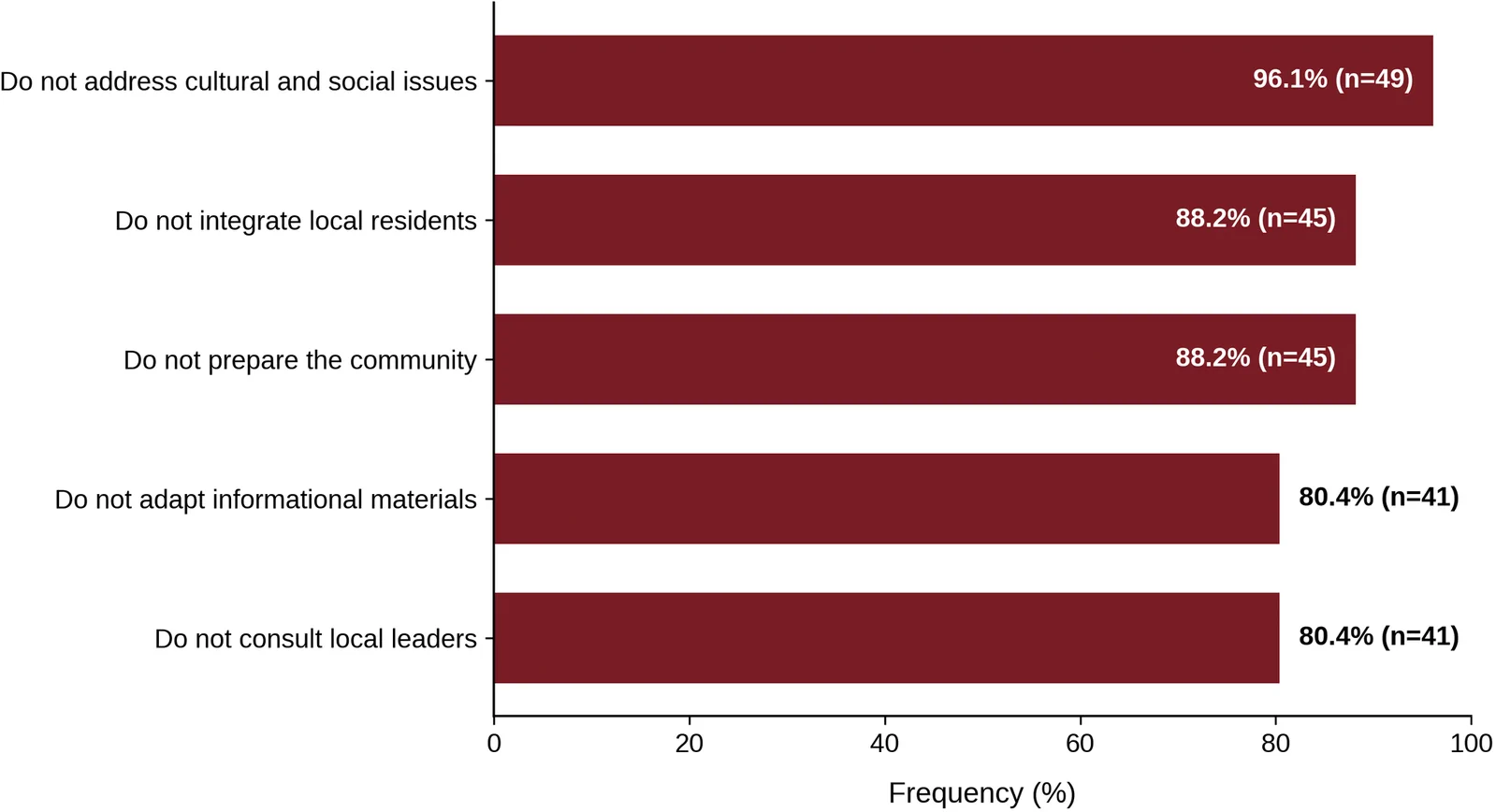
Frequency of inclusion and local adaptation practices not implemented by researchers. Horizontal bars represent the percentage and absolute frequency (n) of participants reporting the non-adoption of specific community engagement and cultural adaptation strategies (*n* = 51).

The adoption of practices focused on inclusion and local adaptation was notably low among participants (*n* = 51). As shown in Fig 3, most respondents (96.1%, *n* = 49) reported that they do not address cultural and social issues in their research activities. Furthermore, 80.4% (*n* = 41) do not adapt informational materials to the local context, and the same percentage (80.4%, *n* = 41) do not consult local leaders. The integration of community residents into the research team and specific community preparation were also rarely implemented, with 88.2% (*n* = 45) of participants reporting a lack of engagement in both categories.

**Fig 4:**
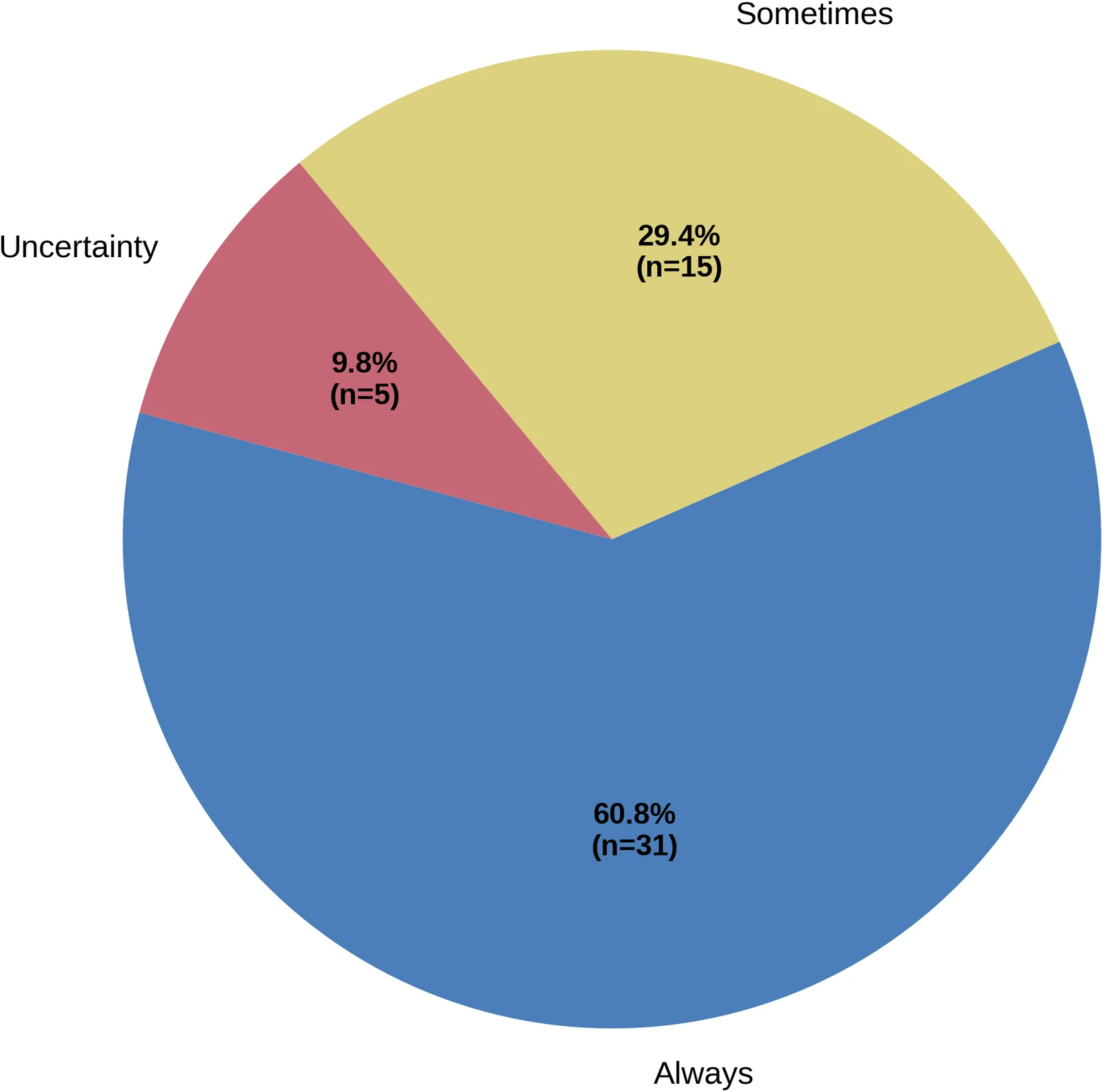
Researchers’ perception of community trust in research findings. Distribution of responses regarding the community’s belief in study findings. Despite low implementation of inclusive practices, the majority of researchers reported high levels of community trust (*n* = 51).

Despite the low implementation of inclusive practices, the community’s perceived level of trust in the research results was high (Fig 4). Most participants (60.8%, *n* = 31) stated that the community “always” believes in the research findings, while 29.4% (*n* = 15) reported that the community trusts the results “sometimes”. Only a small portion of the sample (9.8%, *n* = 5) expressed uncertainty regarding the community’s perception of the validity of the results.

## Discussion

### The geopolitics of scientific capital and demographic asymmetries

The configuration of the Brazilian scientific field, while indicating a transitional scenario marked by a gradual regional decentralization, remains under the aegis of the structural hegemony of the Southeast [12]. This geographic asymmetry converges with the predominance of self-identified white professionals (68.6%) and a high level of educational attainment (80.4% with graduate degrees) observed in the sample, confirming centralized geopolitics of scientific capital that extends beyond the spatial dimension. This scenario corroborates historical patterns in which the Southeast preserves its position as a productive and normative hub, hosting the majority of research groups in contrast to the peripheral representation of the North and Central-West regions [13,14]. In this context, the limited participation of these regions reaffirms the monopoly of scientific authority which, by centralizing funding and recognition, imposes structural barriers to the circulation of cultural capital and the plurality of theoretical perspectives within the national field.

While our sample encompassed research team members across diverse operational and technical roles rather than exclusively principal investigators, the demographic profile observed (68.6% white) resonates with broader structural asymmetries documented in the Brazilian scientific field. For contextual comparison, institutional data on research project leadership at the Federal University of Mato Grosso (UFMT) indicate that white researchers coordinate 86.3% of research projects, while the participation of Black researchers is 2.1% and that of mixed-race researchers is 7.6% [15]. Although direct equivalencies between general field team composition and principal investigator demographics must be drawn with caution, both indicators collectively illustrate that symbolic capital in the Brazilian academic field remains strongly associated with certain demographic profiles, which can create barriers to access and restrict diversity and democratization in knowledge production. The persistence of these geographic and racial concentrations may hinder the incorporation of perspectives aimed at methodological and theoretical diversification, which are essential for fostering plural and inclusive knowledge in public health. This is because this accumulation of symbolic capital acts as a factor that reinforces social distance and a hierarchical relationship between the researcher and the social field under investigation.

When ignoring the influence of racial and class markers on the formation of the scientific *habitus*, actors tend to normalize symbolic violence, in which certain hierarchies of knowledge prevail over local cultural capital. The study suggests that technological democratization and the influx of early-career researchers are still insufficient to subvert the monopoly of symbolic and decision-making power within traditional frameworks. Consequently, the transition toward regionally equitable scientific production remains in its infancy, maintaining the Southeast as the defining hub of research agendas and perpetuating the disparities that science seeks to reduce.

### Structural barriers and the paradox of scientific capital in field research

The challenges of clinical field research in Brazil go beyond operational obstacles, reflecting a social field marked by structural asymmetries in the distribution of scientific and institutional capital. As Lima [16] observes, the field is a relatively autonomous space of practices, yet one permanently traversed by power relations that shape intellectual production.

The high prevalence of logistical obstacles, particularly regarding access to remote locations, reveals that geography acts as a structural barrier that constrains knowledge production and health equity. It is important to emphasize that such obstacles are largely external to the institution, stemming from the precarious state of national infrastructure and regional disparities that lie beyond the institution’s administrative control. As pointed out by Malacarne [17], access to remote areas and indigenous communities is obstructed by a network of impediments in which the high cost of travel restricts scientific production in these regions, perpetuating the exclusion of these populations due to a scarcity of economic and human capital.

In this context, a critical paradox emerges: the precarious nature of scientific work manifests itself in the use of risky modes of transportation, such as small aircraft in the Amazon or travel on hazardous highways. This fact reveals that the symbolic capital and institutional prestige of entities such as Fiocruz do not always translate into the economic capital necessary to ensure the operational safety of teams. Notably, even in projects with adequate financial support, overcoming these challenges is not guaranteed by budgetary resources alone. The complexity of the Brazilian territory imposes physical limits that funding, on its own, cannot overcome, requiring the technical staff to confront vulnerabilities where adaptive “know-how” must compensate for the historical deficit in state investment in safety and basic infrastructure. Conducting science in neglected regions requires the researcher to be, simultaneously, a physician, a logistics manager, a diplomat (to deal with embargoes), and an engineer, operating within a system where nature and the economy constantly work to hinder scientific progress.

This external vulnerability is often exacerbated by meteorological and climatic factors. Climate change not only alters epidemiological patterns but also intensifies structural inequalities [18,19]. In the context of research, extremes in rainfall and temperature threaten the integrity of equipment and the stability of biological samples.

The predominance of women in the sample (76.5%) contrasts with the precarious field conditions, revealing an additional layer of structural vulnerability. The deficient infrastructure of these territories affects female professionals working in the field in a differentiated manner, manifesting itself in extreme situations, such as restricting fluid intake to mitigate dependence on precarious sanitation facilities, Venugopal *et al*. [20]. Furthermore, structural machismo is a persistent phenomenon in the external social sphere, operating as a structure that institutionalizes male hegemony and gender divides. Its most evident manifestation is seen in microaggressions—everyday practices and discourses that, although often normalized [21], constitute decisive barriers to access and retention in the scientific sphere.

In the face of these vulnerabilities and biological risks, contingency management has become a *habitus*, a practical disposition embodied through experience that transcends formal education [22]. This adaptive rigor is the essential response of agents to maintain the integrity of scientific research in the face of climatic and operational threats, ensuring Fiocruz’s excellence even when the external environment proves insufficient to mitigate the real risks of the territory.

### Ethical reflexivity and the dynamics of the scientific field

The ethical challenges identified in this study extend far beyond compliance with regulatory standards. They invite reflection on the subtle power relations and social hierarchies that structure the scientific field [23]. It was observed that research teams tend to attribute ethical dilemmas predominantly to external factors, such as cultural differences. This focus may inadvertently downplay the influence of institutional pressures or internal work dynamics, revealing a “blind spot” that stems from their very immersion in the scientific *habitus*.

Since scientific activity is driven by a legitimate quest for recognition and the accumulation of technical capital, the structures of authority that organize the field can become so deeply ingrained that they operate automatically in everyday practices [9,10,24]. This routinization can create a disconnect between technical excellence and the perception of the asymmetries present in interactions with participants. In this sense, an opportunity arises to promote training that goes beyond normative ethics and encourages critical reflexivity, allowing the researcher to recognize how their position and authority influence the perception and reception of the “other.”

### The path to consent: Dialogue and symbolic connection

The dynamics of social distancing are reflected in practice in the process of obtaining the ICF or Assent Form. The results indicate that this ritual goes beyond an ethical formality, emerging as a moment of connection and, at times, of disconnect driven by language barriers and trust issues. Among researchers who reported difficulties in the consent process, barriers such as participants’ comprehension challenges, misinformation, and mistrust were particularly prominent. These reports suggest that such factors represent decisive bottlenecks for the fluidity of communication between science and the community.

From Bourdieu’s perspective, this reality illustrates how the unequal distribution of cultural and symbolic capital can create a social distance in which the researcher, even without direct intent, is endowed with unquestionable authority. In this scenario, the use of specialized language—identified as an obstacle to understanding in 11 of the reports—may reinforce barriers rather than build bridges. In contexts of vulnerability, the participant’s suppression of doubts—often interpreted as full acceptance—may reflect only a natural inhibition in the face of the symbolic prestige associated with the researcher’s role, which requires careful attention to ensure that consent is, in fact, an act of autonomy.

To harmonize this paradox, strengthening horizontal engagement becomes essential. The researcher plays the inspirational role of mediator in this process, using their cultural capital to translate technical norms into strategies for literacy and dialogue. By co-creating materials with local leaders and adopting more dialogic approaches, the symbolic distance is reduced. By recognizing the participant as a subject possessing legitimate knowledge and experiences, the bureaucratic ritual is transformed into an authentic exercise in partnership, dignity, and social justice.

### Opportunities, engagement, and the construction of legitimacy in the field

Researchers’ perceptions of opportunities in clinical field research reveal a value system that guides scientific practice beyond economic logic, placing the primary gain in the realm of legitimacy and social influence. The most significant opportunities, such as access to specific populations (92.2%), direct impact on the community (90.2%), and contribution to the improvement of public policies (88.2%), indicate that recognition and public utility are the main drivers of value. Thus, according to Bourdieu, by generating tangible social benefits, technical rigor is converted into prestige, transforming into symbolic capital and scientific authority [23]. In this scenario, these opportunities operate as mechanisms for the accumulation of social capital, consolidating ties that form a strategic asset for future research.

### The paradox of engagement and the dissonance of *habitus*

One of the most latent contradictions identified in this diagnosis lies in the disparity between the low implementation of community engagement strategies and the high perception, on the part of researchers, of community’s trust in their results. The data reveal that, although the majority of researchers (96.1%) report not addressing cultural and social issues in their activities, and 80.4% do not consult with local leaders, there is a predominant belief that the community “always” (60.8%) or “sometimes” (29.4%) trusts the research findings. This dissonance suggests the existence of another “blind spot” in the scientific *habitus*, in which the symbolic authority and cultural capital accumulated are often internalized as intrinsic guarantors of credibility. Consequently, in light of the Bourdieusian framework, the hypothesis is raised that this unilateral perception may mask subtle dynamics of symbolic violence, in which the silence or absence of questioning on the part of the community ends up being interpreted by the research teams as full acceptance or unshakable trust, disregarding the latent power asymmetries in the interaction.

The social distance between the agent holding the technical knowledge and the research participant establishes a hierarchy that can inhibit the expression of doubts or mistrust, reinforcing a notion of “neutrality” that, in practice, disregards power asymmetries in the field. Such a belief in unconditional trust, unaccompanied by inclusive practices, indicates that scientific recognition is still operated in a verticalized manner, treating the territory predominantly as an object of data collection rather than a space in which subjects are holders of legitimate cultural capital.

### Shared legitimacy and equity

To overcome historical barriers of mistrust, community engagement must go beyond the role of an ethical safeguard and be framed as a strategy for the redistribution of symbolic capital. By recognizing the population as an active partner, the research team refrains from imposing unilateral knowledge in order to build shared legitimacy [15,25,26]. This approach promotes a horizontal exchange in which the technical staff transfers skills and training, while the community offers its cultural capital and local historical knowledge [25,26]. The inclusion of community members in research teams strengthens the quality of the research, the appropriateness of the materials, and the effectiveness of the interventions [25].

By integrating local knowledge, research gains the depth necessary to understand the specific characteristics of the territory, transforming the *habitus* of researchers and participants through mutual learning. This process enriches methodological rigor and brings about an essential redistribution of cultural capital, promoting the empowerment of communities and reducing the structural inequalities that science aims to address.

The success of clinical field research in Brazil lies, therefore, in the ability to reconcile technical excellence with the generation of direct benefits for society. By transforming the participant into an active subject, science establishes itself as a pillar of public health, contributing to the reduction of inequalities in a complex and diverse national context.

## Conclusions

The results of this study demonstrate that clinical field research at Fiocruz operates at the complex interface between scientific excellence and the structural barriers of vulnerable territories. From Bourdieu’s perspective, the significant prevalence of logistical challenges, cited by 96.1% of participants, along with the identified ethical dilemmas, reflect capital inequalities and disparities of *habitus* between the scientific and social fields. This reality highlights that the consolidation of clinical field research requires transcending a strictly technocratic view in favor of an immersive approach sensitive to local sociocultural dimensions.

In light of these findings, it is imperative to institutionalize logistical and operational risk management plans designed specifically for the unique characteristics of fieldwork. This measure is not merely administrative; it represents the translation of reflexive sociological theory into direct operational improvement for the SUS by integrating the unpredictability of these scenarios into institutional planning. Furthermore, to overcome the “engagement paradox” revealed by the data, strengthening literacy strategies and horizontal dialogic interaction with communities is essential for science to convert its symbolic authority into social capital, establishing itself as a vector for equity and national sovereignty rather than just data collection.

Methodologically, it should be noted that the qualitative component of this mixed-methods study assumes a complementary and exploratory character. It is recognized that qualitative data collection through “other” open-ended fields and voluntary participation may have selected participants with more critical views, potentially limiting the capture of deeper subjectivities. The design relied exclusively on the perceptions of researchers—agents of the scientific field—which prevents a complete triangulation of power relations due to the absence of voices from institutional managers and, crucially, from local community participants. Without the community’s perspective, the trust perceived by the researchers risks being a projection of their own academic *habitus*, failing to detect subtle forms of resistance or symbolic violence.

Finally, the central challenge of a peer-led study must be emphasized: the profound risk of “invisibilities” stemming from the immersion in the field itself. The scientific *habitus* acts as a lens that, by focusing on technical excellence, may naturalize power asymmetries, making them imperceptible in daily professional practice. Even in the face of these structural limitations, by highlighting such tensions and implicit dynamics, this study contributes to a deep reflection on scientific practice. It is recommended that future investigations seek to integrate multiple actors, particularly community leaders, promoting a more plural and symmetrical construction of knowledge in public health. This multiplicity of perspectives can strengthen the design of clinical research, favoring approaches that are more sensitive and responsive to the operational and social realities of vulnerable territories in Brazil and potentially comparable settings.

## Supporting information

S1 Appendix Ethical approval document. Copy of the consolidated opinion from the Research Ethics Committee of the Oswaldo Cruz Institute (Opinion No. 7.698.450).
(PDF)

S2 Appendix Research questionnaire. Full version of the semi-structured data collection instrument hosted on the REDCap platform.
(PDF)

S3 Appendix REDCap data dictionary codebook. Detail of all electronic data collection instruments, including the Informed Consent Form, Principal Investigator Acceptance, and Electronic Questionnaire (PID: 1898), containing variable names, field labels, field types, and validation rules.
(PDF)

S1 Dataset Raw data from the questionnaire. This file contains the complete labeled dataset. The data have been fully de-identified, with all direct and indirect identifiers (e.g., city, date of birth, age, academic background, and profession/job title) removed to protect participant confidentiality.
(CSV)

S1 Fig Hierarchical coding framework for the qualitative. The coding system outlines the thematic axes of challenges (ethical, logistical, and political) and opportunities, alongside mitigation strategies grounded in *habitus* and capital.
(TIF)
