# Supplementary material for: Challenges and opportunities in clinical field research: A Bourdieusian analysis of Fiocruz’s experience in Brazil
Source: PLoS One. 2026 Sep 25;21(9):e0359217. doi: 10.1371/journal.pone.0359217 (PMC13614590; doi:10.1371/journal.pone.0359217)
Supplement: Supplementary file 1 [file pone.0359217.s001.pdf]

FUNDAÇÃO OSWALDO CRUZ – FIOCRUZ / IOC

SUBSTANTIVE OPINION OF THE RESEARCH ETHICS COMMITTEE (CEP)

Researcher: Renata Caldeira Diniz
Research Title: Challenges and Opportunities in Field Clinical Research Projects of the Clinical Research Platform of the Vice-Presidency for Biological Collections and Research at Fiocruz.
Proponent Institution: Vice-Presidency for Research and Biological Collections (VPPCB)
Version: 2
CAAE: 89038525.6.0000.5248
Opinion No.: 7.698.450
Thematic Area: (not specified in the original extract)

Institution: Fundação Oswaldo Cruz (Fiocruz) **Address:** Av. Brasil 4036, room 705 – 7th floor, Campus Maré, Manguinhos, Rio de Janeiro – RJ, Brazil **CEP:** 21.040-361 **Phone:** +55 (21) 3882-9011 **Fax:** +55 (21) 98841-9471 **E-mail:** cepfiocruz@ioc.fiocruz.br

PROJECT RESEARCH DATA

INTRODUCTION

Health research is essential for scientific advancement, improvement of care practices, and the formulation of public policies. It encompasses the study of human biological and psychological processes, the causes and prevention of diseases, and the interactions between environment, society, and health. Law No. 14.874/2024 regulates research involving human beings in Brazil, including clinical investigations and the use of personal or biological data, reinforcing its central role in the development of treatments and preventive strategies. Despite progress, challenges persist and require the continuous production of knowledge adapted to social and cultural realities.

STUDY DESIGN

The proposed research is qualitative and is guided by Pierre Bourdieu’s social theory, based on the concepts of field, habitus, and symbolic capital. Bourdieu proposes a reflexive sociology, which requires the researcher to adopt a critical stance regarding their position in the field, advocating the choice of methods appropriate to the characteristics of the object of study. The study population will consist of researchers and team members involved in six projects supported by the Clinical Research Platform/VPPCB between 2020 and 2023 (PCL 01/20, PCL 01/23, PCL 02/23, PCL 03/23, PCL 04/23, PCL 05/23). Selection will be by convenience, including participants who voluntarily agree to participate and sign the Informed Consent Form (ICF/TCLE). An estimated sample of approximately 50 participants is expected. The data collection instrument will be a semi-structured questionnaire.

METHODOLOGY

The research adopts a qualitative approach, appropriate for understanding complex human phenomena, especially in the field of health, integrating knowledge from biological and social sciences. The theoretical framework adopted is Pierre Bourdieu's reflexive sociology, focusing on the concepts of field, habitus, and symbolic capital, which guide the analysis of researchers' practices in the context of field clinical research. The target population will be composed of researchers and professionals involved in six projects supported by the Clinical Research Platform/VPPCB between 2020 and 2023. The sample will be one of convenience, estimated at around 50 participants, with inclusion criteria related to having worked on the projects for at least 1 month. Data collection will be carried out through a semi-structured, self-administered, online, anonymous, and voluntary questionnaire, developed on the REDCap platform. The instrument will contain five sections: participant characterization; field research challenges; ethics and quality; opportunities; and understanding of clinical research. The questionnaire construction in REDCap will involve the use of different field types available, such as multiple-choice questions, text boxes for open-ended responses, and "other" options. Branching logic (conditional display) will also be implemented, so that certain questions are shown only according to previous answers, making completion smoother, more dynamic, and personalized for each participant.

RESEARCH OBJECTIVES

Primary objective: To analyze the main challenges and opportunities faced by researchers and other professionals involved in field clinical research supported by the Platform of the Vice-Presidency for Research and Biological Collections (VPPCB), based on the portfolio of clinical research projects from 2020 and 2023.

Secondary objectives:
1. To conduct a SWOT analysis (Strengths, Weaknesses, Opportunities and Threats) related to the implementation and execution of field clinical research supported by the Platform/VPPCB/Fiocruz, in order to support the development of strategies that strengthen strengths, minimize weaknesses, seize opportunities, and mitigate identified risks.
2. To draft a proposed Work Instruction (WI/IT) focused on the collection and management of adverse events in field clinical research (Work Instruction – Collection and Management of Adverse Events in Field Clinical Research Projects), with guidelines adapted to the specificities of this type of study, aiming to ensure accuracy in documentation, participant safety, and compliance with ethical and regulatory requirements.
3. To draft a proposed Risk Management Plan template that addresses the inherent specificities of field clinical research (Risk Management Plan for Field Clinical Research Projects).

RISK AND BENEFIT ASSESSMENT

Benefits: Participation in this project may provide those involved with an opportunity to critically reflect on their performance in field clinical research projects, in addition to contributing to the improvement of Fiocruz institutional practices. Participants may identify gaps, challenges, and potentialities in their work contexts, favoring the development of competencies and valuing their experiences. It is also expected that the project results will support training actions and the customization of the existing processes of the VPPCB Clinical Research Platform to the specificities required by field clinical research, directly benefiting the professionals involved and, indirectly, the research projects in which they work.

Risks: This project presents minimal risks to participants, since it does not involve clinical interventions, collection of sensitive health data, or invasive procedures. However, it is possible that some participants may feel discomfort when reflecting on or answering questions related to challenges faced in their work in field clinical research project(s), especially when addressing aspects related to quality, ethics, or institutional and operational issues.

COMMENTS AND CONSIDERATIONS ON THE RESEARCH

This is a well-founded project, with no need to be forwarded to CONEP. Study type: Qualitative. Participant recruitment will be carried out in six Clinical Research projects developed by the Vice-Presidency for Research and Biological Collections (Fiocruz). Sample size in Brazil: 50. Expected start date: Jun/2025. Invitation/Recruitment: Jul/2025. Expected end date: Dec/2025. Budget: R$ 1,500.00. Participating institutions: VPPCB – Fiocruz.

All required files were submitted. New documents were added to the protocol:
- TCLE_Ambiente_Virtual_com_alteracoes_destacadas_versao_2_20250626 (DOCX)
- TCLE_Ambiente_Virtual_versao_2_20250626 (PDF)
- Carta_Resposta_ao_Parecer_7664331_20250627 (PDF)

Findings were compliant and approved.

CONSIDERATIONS ON MANDATORY SUBMISSION TERMS

Analysis of the responses to Substantive Opinion No. 7,664,331 of this CEP/IOC: No ethical obstacles were observed for the protocol under review.

CONCLUSIONS / PENDING ITEMS AND LIST OF INADEQUACIES

Single pending item – TCLE_Ambiente_Virtual_versao_1_20250505.pdf

1.1 Inform, in the ICF/TCLE or in the Consent Record, in a highlighted manner, the importance of the participant keeping, in their files, a copy of the document and/or ensuring that the researchers send the signed copy to the research participant.

Response: As requested, the guidance regarding the importance of the participant storing a copy of the document was included in a highlighted manner on page 3 of the ICF/TCLE. The inserted text was: "You will receive your copy of the ICF/TCLE duly signed by e-mail. You must store, in your files, a printed or digital copy of the document." In this sense, this information ensures that the participant is aware of the need to keep a copy of the Informed Consent Form, meeting the principle of transparency and the right to information.

ANALYSIS: PENDING ITEM ADDRESSED.

1.2 By underestimating the risks involved in a study, the researcher does not convey the information necessary for the individual to make an autonomous decision about participating in the research. This also prevents the researcher from anticipating ways and mechanisms to minimize risks, increasing the risk of harm to participants and violating the ethical principle of prudence. Please remove the expression "This project involves minimal risks." (p. 2).

Response: In compliance with the guidance regarding project risks, the expression "This project involves minimal risks" was removed from page 2. The excerpt was revised to: "The risks of this project may be related to discomfort…" This change aims to avoid underestimation of risks and reinforces the ethical commitment to prudence, promoting a more informed and autonomous decision by the participant.

ANALYSIS: PENDING ITEM ADDRESSED.

FINAL CONSIDERATIONS (AT THE DISCRETION OF THE CEP)

In view of the above, the Research Ethics Committee of the Oswaldo Cruz Institute (CEP Fiocruz/IOC) expresses its approval of the proposed research project. The researcher must guarantee the research participant the freedom to refuse to participate or to withdraw consent at any stage of the research, without any penalty and without prejudice to their care, and must provide the participant with a complete version of the Informed Consent Form (ICF/TCLE), signed and dated by the participant and by the responsible team member. The researcher must conduct the research as outlined in the approved protocol. This CEP emphasizes the importance of submitting annual partial reports and the final report, which is one of the responsibilities assumed by the researcher when submitting the project for review.

According to Law 14.874/2024, Art. 27, the responsibilities of the researcher are: (I) to have and prove the qualifications and experience necessary to assume responsibility for the proper conduct of the research; (II) to comply with standards of good clinical and scientific practices and regulatory requirements; (III) to submit research documentation, including any amendments, to CEP approval; (IV) to select qualified persons to perform research-related tasks; (V) to ensure, in the case of a clinical trial, the clinical follow-up of research participants during the conduct of the study and after its end, for the period and under the conditions defined in the protocol approved by the CEP; (VI) to conduct the research in accordance with the project approved by the CEP; (VII) to provide, when requested, direct access to research records and documents to the monitor, auditor, other sponsor representatives, the CEP, the national research ethics authority, and the health authority; (VIII) to allow monitoring and auditing of the research by the sponsor, as well as inspections by the health authority, the national research ethics authority, and the CEP that approved it; (IX) to submit partial reports with information on research progress, annually and whenever requested, to the CEP that analyzed it; (X) to promptly communicate to the sponsor, the health authority, the CEP, and the national research ethics authority all serious or unexpected adverse events; (XI) to keep stored and under their custody, in physical or digital form, the data and essential research documents for a period of 5 (five) years after its completion or formal discontinuation, and for 10 (ten) years in the case of advanced therapy products; (XII) to ensure the rights of research participants and safeguard their well-being and safety.

It should be noted that Art. 60 of the aforementioned Law, in force since August 2024, provides that noncompliance with the provisions of Arts. 26, 27, 35, 42, 51 and 55 of this Law and failure to comply with Good Clinical Practice (GCP) standards, as set forth in the regulation, constitutes a sanitary infraction and subjects the offender to the penalties provided in Law No. 6,437, of 20 August 1977, and in specific sanitary regulations, without prejudice to applicable civil and criminal sanctions.

THIS OPINION WAS PREPARED BASED ON THE DOCUMENTS LISTED BELOW

Document Type	File	Upload Date/Time	Author	Status
Basic Project Information	PB_INFORMACOES_BASICAS_DO_PROJETO_2556629.pdf	27/06/2025 19:54:24	-	Accepted
ICF/TCLE / Assent Terms / Justification of Absence	TCLE_Ambiente_Virtual_versao_2_alteracoes_destacadas_20250626.doc	27/06/2025 19:53:03	Renata Caldeira Diniz	Accepted
ICF/TCLE / Assent Terms / Justification of Absence	TCLE_Ambiente_Virtual_versao_2_20250626.pdf	27/06/2025 19:51:40	Renata Caldeira Diniz	Accepted
Other	Carta_Resposta_ao_Parecer_7664331_20250627.pdf	27/06/2025 19:50:52	Renata Caldeira Diniz	Accepted
ICF/TCLE / Assent Terms / Justification of Absence	TCLE_Ambiente_Virtual_versao_1_20250505.pdf	26/05/2025 17:00:39	Renata Caldeira Diniz	Accepted
Detailed Project / Investigator Brochure	Projeto_de_Pesquisa_versao_1_20250505.pdf	26/05/2025 16:53:24	Renata Caldeira Diniz	Accepted
Budget	Orcamento_do_Projeto_versao_1_20250505.pdf	26/05/2025 16:51:04	Renata Caldeira Diniz	Accepted
Other	Modelo_de_Convite_E_mail_Agradecimento_versao_1_20250505.pdf	23/05/2025 22:36:51	Renata Caldeira Diniz	Accepted
Other	Curriculo_Lattes_Natalia_Moraes.pdf	23/05/2025	Renata Caldeira Diniz	Accepted
Other	Curriculo_Lattes_Karla_Gram.pdf	23/05/2025 22:35:02	Renata Caldeira Diniz	Accepted
Other	Curriculo_Lattes_Jocieli_Malacarne.pdf	23/05/2025 22:34:39	Renata Caldeira Diniz	Accepted
Other	Curriculo_Lattes_Cassia_Senra.pdf	23/05/2025 22:34:24	Renata Caldeira Diniz	Accepted
Other	Carta_Submissao_20250523.pdf	23/05/2025 22:34:09	Renata Caldeira Diniz	Accepted
Researchers' Statement	Declaracao_Concordancia_Pesquisadora_Responsavel_20250523.pdf	23/05/2025 22:33:22	Renata Caldeira Diniz	Accepted
Researchers' Statement	Declaracao_Pesquisadora_Colaboradora_20250518.pdf	23/05/2025 22:26:42	Renata Caldeira Diniz	Accepted
Agreement Statement	Declaracao_Concordancia_Pesquisadoras_20250523.pdf	23/05/2025 22:26:20	Renata Caldeira Diniz	Accepted
Researchers' Statement	Declaracao_Pesquisadora_Responsavel_20250516.pdf	23/05/2025 22:22:06	Renata Caldeira Diniz	Accepted
Other	Curriculo_Lattes_Renata_Diniz.pdf	23/05/2025 21:40:18	Renata Caldeira Diniz	Accepted
Other	Questionario_versao_1_20250505.pdf	23/05/2025 21:26:31	Renata Caldeira Diniz	Accepted
Institution & Infrastructure Statement	Declaracao_Infraestrutura_20250516.pdf	23/05/2025 21:24:31	Renata Caldeira Diniz	Accepted
Institutional Consent Statement	Declaracao_Anuencia_Institucional_SEI_5022894_Oficio_146_Dra_Maria_Lourdes_20250522.pdf	23/05/2025 21:24:13	Renata Caldeira Diniz	Accepted
Schedule	Cronograma_do_Projeto_versao_1_20250505.pdf	23/05/2025 21:23:54	Renata Caldeira Diniz	Accepted

Cover Sheet	Folha_Rosto_Assinada_SEI_5023148_Oficio_147.pdf	23/05/2025 21:20:55	Renata Caldeira Diniz	Accepted

Rio de Janeiro, 09 July 2025
Signed by: Ximena Illarramendi (Coordinator)

Opinion status: Approved
Requires CONEP review: No

Translator's note: This English version is a faithful translation of the textual content extracted from the provided PDF. Logos, decorative elements, and blank table layouts present in the original were not reproduced; all substantive text was translated while preserving acronyms and file names.
