# Supplementary material for: Challenges and opportunities in clinical field research: A Bourdieusian analysis of Fiocruz’s experience in Brazil
Source: PLoS One. 2026 Sep 25;21(9):e0359217. doi: 10.1371/journal.pone.0359217 (PMC13614590; doi:10.1371/journal.pone.0359217)
Supplement: Supplementary file 2 [file pone.0359217.s002.pdf]

ELECTRONIC QUESTIONNAIRE

Please complete the survey below.

Thank you!

PARTICIPANT AND RESEARCH CHARACTERIZATION

User responsible for completion: ______________________________

Date and time of completion: ______________________________

ELIGIBILITY CRITERIA

Are you 18 years old or older?
- ◯ Yes
- ◯ No

Have you been working for more than a year on at least one of the following projects (PCL 01/20: qPCR for TB; PCL 01/23: Saneaxisto; PCL 02/23: PRAped; PCL 03/23: Validaxisto; PCL 04/23: Oxente; PCL 05/20: DIACHO)?
- ◯ Yes
- ◯ No

- ☐ PCL 01/20: qPCR para TBc
- ☐ PCL 01/23: Saneaxisto
- ☐ PCL 02/23: PRAped
- ☐ PCL 03/23: Validaxisto
- ☐ PCL 04/23: Oxente
- ☐ PCL 05/23: DIACHO

Note: Thank you for your participation, but at the moment you do not meet the minimum criteria to answer this survey.

PARTICIPANT CHARACTERIZATION

State (UF):
- ◯ Acre
- ◯ Alagoas
- ◯ Amazonas
- ◯ Amapá
- ◯ Bahia
- ◯ Ceará
- ◯ Distrito Federal
- ◯ Espírito Santo
- ◯ Goiás
- ◯ Maranhão
- ◯ Minas Gerais
- ◯ Mato Grosso do Sul
- ◯ Mato Grosso
- ◯ Pará
- ◯ Paraíba
- ◯ Pernambuco
- ◯ Piauí
- ◯ Paraná
- ◯ Rio de Janeiro
- ◯ Rio Grande do Norte
- ◯ Rondônia
- ◯ Roraima
- ◯ Rio Grande do Sul
- ◯ Santa Catarina
- ◯ Sergipe
- ◯ São Paulo
- ◯ Tocantins

City ________________

Date of Birth: ________________

Sex
◯ Female ◯ Male
◯ Prefer not to answer

Self-declared Color/Race:
◯ Branca ◯ Black ◯ Mixed
◯ Amarela ◯ Indigenous
◯ Prefer not to answer

Age: ________________

Education Leve
◯ Higth School
◯ Completed Undergraduate
◯ Incomplete Undergraduate
◯ Completed Postgraduate
◯ Incomplete Postgraduate
◯ Prefer not to answer

What is your academic background/degree? ________________

RESEARCH ROLES

What is/are your role(s) in the research?
(Check all that apply):

- ☐ Regulatory Affairs Analyst
- ☐ Data Analyst
- ☐ Quality Analyst
- ☐ Pharmacovigilance Analyst
- ☐ Data Assistant
- ☐ Quality Assistant
- ☐ Research Assistant
- ☐ Coordinator
- ☐ Responsible Co-Researcher
- ☐ Nurse
- ☐ Pharmacist
- ☐ Research Manager
- ☐ Clinical Monitor
- ☐ Principal Investigator
- ☐ Sub-investigator
- ☐ Nursing Technician
- ☐ Laboratory Technician
- ☐ Other
- ☐ Prefer not to answer

Other role held (specify): ________________

FIELD RESEARCH EXPERIENCE

Field health research refers to research conducted outside of controlled environments, such as hospitals, research centers, and laboratories, and is therefore conducted directly in locations where participants live, work, and/or frequent.

Do you work or have you ever worked with field health research projects?
◯ Yes ◯ No

How many field health research projects have you worked on?
◯ 1 a 4 ◯ 5 a 9 ◯ More than 10

How many years of experience do you have with **field health research projects?**

- ◯ 1 a 4 years
- ◯ 5 a 9 years
- ◯ 10 a 14 years
- ◯ 15 a 24 years
- ◯ 25 a 29 years
- ◯ Mais de 30 years

What type of health research do you work on or have you worked on in the field? (Check all that apply)

- ☐ Research on the effects of factors or health states (lifestyle habits, environmental or genetic conditions, and health outcomes).
- ☐ Experimental research with one or more human beings conducted to evaluate the safety, clinical performance, or efficacy of a medical device, experimental drug, or advanced therapy.
- ☐ Health products (diagnostic tests, laboratory tests, or imaging) for diagnostic purposes.
- ☐ Verifying the distribution of risk factors, diseases, or health conditions in the population.
- ☐ Other

Other (specify): ______________________________

Health research may involve data collection in two ways: direct or indirect.

The direct method occurs when there is interaction with the participant, such as in interviews, questionnaire administration, focus groups, in-person observations, blood collection, physical exams, clinical tests, or any other type of contact with the individual to obtain data or biological material.

The indirect method happens when data, documents, images, or biological materials previously collected are used without direct contact with the participant, even if they can be identified. This includes the analysis of medical records, access to databases, previously performed tests, use of stored biological material, as well as images, recordings, or public/private information that allows for the identification of the participant.

Which data collection method have you worked with, directly or indirectly?

◯ Direct ◯ Indirect ◯ Both

FIELD RESEARCH CHALLENGES

Guidance for completion: The following questions refer to the challenges faced. Please respond considering exclusively the field health research project in which you work, which belongs to the portfolio of the VPPCB/Fiocruz Clinical Research Platform.

Do you consider that the field health research project has challenges?

◯ Yes ◯ No

Which challenges in the field health research project do you consider most relevant?

- ☐ Climatic/Meteorological
- ☐ Logistical (local infrastructure, transport, etc.)
- ☐ Ethical (consent, confidentiality, community impact, etc.)
- ☐ Data quality/Information collection
- ☐ Other

(Check all that apply)

Other (specify): __________________________________

What logistical challenges have you faced or do you face when conducting the field health research project?

- ☐ Access to remote locations
- ☐ Team accommodation
- ☐ Team meals/food
- ☐ Storage of source documents / Essential documents
- ☐ Ineffective communication with local teams
- ☐ Difficulty in obtaining permissions or authorizations
- ☐ Unforeseen issues with equipment or materials
- ☐ Limited time for executing activities
- ☐ Logistical costs
- ☐ Constant lack of electricity
- ☐ Lack of health infrastructure
- ☐ Lack of basic sanitation
- ☐ Supply and inventory management
- ☐ Safety and operational risks
- ☐ Transport
- ☐ Transport of biological samples
- ☐ Other

(Check all that apply)

Other logistical challenge (specify): __________________________________

What ethical challenges have you faced or do you face when conducting the field health research project?

- ☐ Social, cultural, and value differences.
- ☐ Ensuring participant anonymity and confidentiality.
- ☐ Impact of the research on local communities.
- ☐ Obtaining the Informed Consent Form (ICF) or Assent Form.
- ☐ Institutional pressures or conflicts of interest.
- ☐ Power dynamic between researcher and participant.
- ☐ Use and sharing of collected data.
- ☐ Other

(Check all that apply)

Other challenge(s) in obtaining the ICF or Assent Form (specify): __________________________________

Challenges in obtaining the ICF or Assent Form:

- ☐ Language barriers
- ☐ Mistrust
- ☐ Participant misinformation
- ☐ Difficulty in understanding
- ☐ Difficulty in communicating research objectives and methods
- ☐ Other

Other ethical challenge(s) (specify): __________________________________

How do you address cultural and social issues when conducting the field health research project?

☐ I do not address them
☐ I adapt informative materials to the local culture
☐ I consult local leaders to review the best language for the target population
☐ I integrate local residents into the research team
☐ I prepare the community with pedagogical actions that promote critical thinking and awareness of research procedures and risks, allowing for an informed decision about participation.
☐ Other
(Check the two main approaches)

Other cultural and social issues (specify): ______________________________

ETHICS AND QUALITY

The following questions refer to quality, ethics, and participant safety. Please respond considering exclusively the field health research project in which you work, which belongs to the portfolio of the VPPCB/Fiocruz Clinical Research Platform.

Do you believe that the participants recruited in the field research project are/were sufficiently informed about the risks and benefits of the study? ○ Yes ○ No

---

In the field research project, was an Informed Consent Form (ICF) and/or Assent Form obtained? ○ Yes ○ No

Do you record the consent process in the project's source document? (the application of an assent form and/or informed consent form)

◯ Yes ◯ No

How do/did you record the consent process?

☐ Description in the medical progress notes of the institution's record
☐ Pre-filled form
☐ Checklist
☐ Note to File or similar
☐ Other

Other (specify):

____

According to Law No. 14,874, of May 28, 2024, which regulates research involving human beings and establishes the National Ethics System for Research Involving Human Beings, an Adverse Event (AE) is any unfavorable medical occurrence, whether or not related to the product under investigation, in a patient or research participant during its execution.

Do/did you collect and report Adverse Events occurring with participants in the field research project?

◯ Yes ◯ No ◯ Not applicable to the project

According to Law No. 14,874, of May 28, 2024, which regulates research involving human beings and establishes the National Ethics System for Research Involving Human Beings, a Serious Adverse Event (SAE) is any unfavorable medical occurrence that results in: death, life-threatening risk, situations requiring hospitalization or prolongation of hospitalization, significant disability, congenital anomaly, or a clinically significant event.

In case of a Serious Adverse Event in the field research project, do you collect the SAE and report it to the Sponsor and the Research Ethics Committee?

◯ Yes ◯ No ◯ Not applicable to the project

Medical resources necessary for conducting field health research are the means, equipment, and structures that ensure the safety and well-being of participants and the research team. They are essential for preventing risks and allowing for care in emergency cases. These resources vary according to the type and location of the research and include: first aid kits, PPE (Personal Protective Equipment), an appropriate site for biological waste disposal, emergency action plans, prior knowledge of nearby health facilities, and emergency communication methods in remote areas.

Do you consider having/had adequate access to the medical resources necessary to conduct the field health research project?

◯ Yes ◯ No ◯ Not applicable to the project

Does your research center have Standard Operating Procedures (SOPs) for conducting the field esearch project?

- ☐ Contingency Plan
- ☐ IRB system procedures
- ☐ Disposal of biological and non-biological materials
- ☐ Team training and retraining
- ☐ Recruitment and selection of research participants
- ☐ Consent process and obtaining the ICF
- ☐ Verification of source documents and CRFs
- ☐ Use and calibration of equipment/instruments
- ☐ Collection, transport, preparation, identification, and analysis of laboratory samples
- ☐ Receipt, accounting, destruction, or return of the investigational product
- ☐ Does not have any of the mentioned

(Check all that apply)

Are there established Standard Operating Procedures (SOPs) exclusively for the procedures involved in the execution of the field research project?

◯ Yes ◯ No ◯ Not applicable to the project

List the main ones:

____

How do you ensure the quality of the data collected during field health research?

- ☐ I do not ensure it
- ☐ Team training
- ☐ Double-checking completed source documents
- ☐ Review of clinical forms
- ☐ Constant supervision to ensure standardization and information consistency
- ☐ I use validated instruments
- ☐ I use validated electronic clinical forms (e.g., OpenClinic®, REDCap®, etc.)
- ☐ Other

(Check the two main actions)

Other quality assurance action:

____

Do you follow a research protocol or plan when conducting your fieldwork?

- ◯ Yes, I always follow a formal pre-established
- ◯ Sometimes, I follow a protocol, but with flexibility
- ◯ No, I do not follow a formal protocol
- ◯ I am not sure

OPORTUNIDADES DA PESQUISA DE CAMPO

The following questions refer to opportunities in field research projects. Please respond considering exclusively the field health research project in which you work, which belongs to the portfolio of the VPPCB/Fiocruz Clinical Research Platform.

In your assessment, what are the main opportunities identified within the scope of the field health research project?

- ☐ Access to a specific population
- ☐ Adaptation to local conditions (cultural, social, economic)
- ☐ Collection of more representative data
- ☐ Direct impact on the community
- ☐ Institutional incentives
- ☐ Improvement in Public Health Policies
- ☐ Learning opportunities from the local population
- ☐ Other

(Check all that apply)

Other (specify): ______________________________

In your perspective, what is the potential impact of the field health research project on the local community?

- ☐ Access to innovative treatments
- ☐ Education about diseases
- ☐ Involving communities in decision-making regarding health needs and treatments
- ☐ Improvement in the population's health
- ☐ No impact
- ☐ Other

(Check all that apply)

Other impact (specify): ______________________________

Do you believe that participants in the field health research project demonstrate trust in the results generated by the research?

- ◯ Yes, always
- ◯ Sometimes
- ◯ No
- ◯ I am not sure

How do you handle the disclosure of health research project results to participants?

- ◯ I do not disclose them
- ◯ Distributing accessible reports (e-mail, social media, etc.)
- ◯ Offering direct feedback
- ◯ Holding community meetings
- ◯ Other

Other (specify): ______________________________

CLINICAL RESEARCH

For the following questions, please provide your response based exclusively on your professional experience.

Do you consider field health research to be clinical research?

- ◯ Yes
- ◯ No
- ◯ I am not sure
- ◯ Prefer not to answer

Have you ever participated in any course or training in clinical research?
(Check all that apply):

- ☐ Long-term course (e.g., Master's, PhD, specialization)
- ☐ Short-term course (e.g., workshops, seminars)
- ☐ Formal training (courses, certifications, etc.)
- ☐ Informal training (mentoring, practical guidance)
- ☐ Informal training (during a Clinical Research Project Initiation Monitoring Visit)
- ☐ I have never participated

(Check all that apply)

Was the Clinical Research Project Initiation Monitoring Visit conducted by the Clinical Research Platform of VPPCB/Fiocruz?

◯ Yes ◯ No

Do you believe that the lack of clinical research training influences the understanding of what characterizes clinical research?

- ◯ Yes, it directly influences it
- ◯ No, it does not influence it
- ◯ I am not sure
- ◯ Prefer not to answer

Do you believe your institution's culture favors or hinders the understanding of clinical research?

- ◯ Favors, as there is a strong emphasis on defining and practicing clinical research
- ◯ Hinders, as there is no clear differentiation between clinical research and other types of research
- ◯ I am not sure
- ◯ Prefer not to answer
