# Supplementary material for: Challenges and opportunities in clinical field research: A Bourdieusian analysis of Fiocruz’s experience in Brazil
Source: PLoS One. 2026 Sep 25;21(9):e0359217. doi: 10.1371/journal.pone.0359217 (PMC13614590; doi:10.1371/journal.pone.0359217)
Supplement: Supplementary file 3 [file pone.0359217.s003.pdf]

Data Dictionary Codebook – English Translation

Challenges and Opportunities in Field Clinical Research Projects of the VPPCB Clinical Research Platform at Fiocruz (PID: 1898)

This PDF contains the translated REDCap data dictionary fields (variables 26–94). Variable names/codes were kept as in REDCap.

No.	Variable	Field label (EN)	Attributes / branching logic / options (EN)
26	[ie_username]	Section header: PARTICIPANT AND RESEARCH CHARACTERIZATION. User responsible for completing the form	Field type: text. Required. Annotations: @HIDDEN @USERNAME
27	[ie_versao_now]	Date and time of completion	Field type: text. Required. Annotations: @HIDDEN @NOW
28	[ie_idade_yn]	Section header: ELIGIBILITY CRITERIA. Are you 18 years old or older?	Field type: radio. Required. Options:<br>1: Yes<br>0: No<br>Custom alignment: RH
29	[ie_projetos_yn]	Have you worked for more than one year on at least one of the projects (PCL 01/20: qPCR for TBc; PCL 01/23: Saneaxisto; PCL 02/23: PRAped; PCL 03/23: Validaxisto; PCL 04/23: Oxente; PCL 05/20: DIACHO)?	Field type: radio. Required. Options:<br>1: Yes<br>0: No
30	[ie_projetos_qual]	Which project(s) do you work on or have you worked on?	Show field ONLY if: [ie_projetos_yn] = 1 AND [ie_idade_yn] = 1.<br>Field type: checkbox. Required. Options:<br>1: PCL 01/20: qPCR for TBc<br>2: PCL 01/23: Saneaxisto<br>3: PCL 02/23: PRAped<br>4: PCL 03/23: Validaxisto<br>5: PCL 04/23: Oxente<br>6: PCL 05/23: DIACHO
31	[text1]	Thank you for your participation, but at this moment you do not meet the minimum criteria to answer this survey.	Show field ONLY if: [ie_projetos_yn] = 0 OR [ie_idade_yn] = 0.<br>Field type: descriptive

32	[dm_estado]	Section header: PARTICIPANT CHARACTERIZATION. State (UF)	Show field ONLY if: [ie_projetos_yn] = 1 AND [ie_idade_yn] = 1.<br>Field type: dropdown. Required. Options:<br>AC: Acre<br>AL: Alagoas<br>AM: Amazonas<br>AP: Amapá<br>BA: Bahia<br>CE: Ceará<br>DF: Federal District<br>ES: Espírito Santo<br>GO: Goiás<br>MA: Maranhão<br>MG: Minas Gerais<br>MS: Mato Grosso do Sul<br>MT: Mato Grosso<br>PA: Pará<br>PB: Paraíba<br>PE: Pernambuco<br>PI: Piauí<br>PR: Paraná<br>RJ: Rio de Janeiro<br>RN: Rio Grande do Norte<br>RO: Rondônia<br>RR: Roraima<br>RS: Rio Grande do Sul<br>SC: Santa Catarina<br>SE: Sergipe<br>SP: São Paulo<br>TO: Tocantins
33	[dm_cidade]	City	Show field ONLY if: [ie_projetos_yn] = 1 AND [ie_idade_yn] = 1.<br>Field type: text. Required
34	[dm_nascimento]	Date of birth	Show field ONLY if: [ie_projetos_yn] = 1 AND [ie_idade_yn] = 1.<br>Field type: text (date_dmy). Required
35	[dm_sexo]	Sex	Show field ONLY if: [ie_projetos_yn] = 1 AND [ie_idade_yn] = 1.<br>Field type: radio. Required. Options:<br>1: Female<br>2: Male<br>9: Prefer not to answer
36	[dm_cor]	Self-declared race/color	Show field ONLY if: [ie_projetos_yn] = 1 AND [ie_idade_yn] = 1.<br>Field type: dropdown. Required. Options:<br>1: White<br>2: Black<br>3: Brown (Parda/Mixed)<br>4: Yellow (Asian)<br>5: Indigenous<br>95: Prefer not to answer
37	[dm_idade]	Age	Show field ONLY if: [ie_projetos_yn] = 1 AND [ie_idade_yn] = 1.<br>Field type: text (integer). Required. Custom alignment: RH

38	[dm_escolaridade]	Education level	Show field ONLY if: [ie_projetos_yn] = 1 AND [ie_idade_yn] = 1.<br>Field type: radio. Required. Options:<br>1: High school<br>2: Completed undergraduate degree<br>3: Incomplete undergraduate degree<br>4: Completed postgraduate degree<br>5: Incomplete postgraduate degree<br>95: Prefer not to answer
39	[dm_formacao]	What is your academic background/training?	Show field ONLY if: [ie_projetos_yn] = 1 AND [ie_idade_yn] = 1.<br>Field type: text. Required
40	[dm_cargo]	Section header: ROLES IN THE RESEARCH. Which role(s) do you hold in the research (select all that apply):	Show field ONLY if: [ie_projetos_yn] = 1 AND [ie_idade_yn] = 1.<br>Field type: checkbox. Required. Options:<br>1: Regulatory Affairs Analyst<br>2: Data Analyst<br>3: Quality Analyst<br>4: Pharmacovigilance Analyst<br>5: Data Assistant<br>6: Quality Assistant<br>7: Research Assistant<br>8: Coordinator<br>9: Co-Principal Investigator<br>10: Nurse<br>11: Pharmacist<br>12: Research Manager<br>13: Clinical Monitor<br>14: Principal Investigator<br>15: Subinvestigator<br>16: Nursing Technician<br>17: Laboratory Technician<br>99: Other<br>95: Prefer not to answer<br>Annotations: @NONEOFTHEABOVE = 95
41	[dm_cargo_outro]	Other role held (specify):	Show field ONLY if: [dm_cargo(99)] = 1.<br>Field type: text. Required
42	[text2]	Section header: FIELD RESEARCH EXPERIENCE. Field health research refers to research conducted outside controlled environments, such as hospitals, research centers, and laboratories, and therefore conducted directly in the places where participants live, work, and/or attend.	Show field ONLY if: [ie_projetos_yn] = 1 AND [ie_idade_yn] = 1.<br>Field type: descriptive
43	[qs_trabalha_campo]	Do you work or have you worked with field health research projects?	Show field ONLY if: [ie_projetos_yn] = 1 AND [ie_idade_yn] = 1.<br>Field type: radio. Required. Options:<br>1: Yes<br>0: No
44	[qs_projetos_qtd]	How many field health research projects have you worked on?	Show field ONLY if: [qs_trabalha_campo] = 1.<br>Field type: radio. Required. Options:<br>1: 1 to 4<br>2: 5 to 9<br>3: More than 10

45	[qs_anos_experiencia]	How many years of experience do you have with field health research projects?	Show field ONLY if: [qs_trabalha_campo] = 1.<br>Field type: radio. Required. Options:<br>1: 1 to 4 years<br>2: 5 to 9 years<br>3: 10 to 14 years<br>4: 15 to 24 years<br>5: 25 to 29 years<br>6: More than 30 years
46	[qs_tp_pesquisa]	What type of health research do you work on or have you worked on in the field? (Select all that apply)	Show field ONLY if: [qs_trabalha_campo] = 1.<br>Field type: checkbox. Required. Options:<br>1: Studies on effects of risk factors or health states (lifestyle habits, environmental or genetic conditions) and health outcomes.<br>2: Experimental research with one or more humans conducted to evaluate the safety, clinical performance, or efficacy of a medical device, investigational drug, or advanced therapy.<br>3: Health products (diagnostic tests, laboratory tests, or imaging tests) for diagnostic purposes.<br>4: Assess the distribution of risk factors, diseases, or health conditions in the population.<br>99: Other<br>Custom alignment: LV
47	[qs_tp_pesquisa_outro]	Other (specify):	Show field ONLY if: [qs_tp_pesquisa(99)] = 1.<br>Field type: text. Required
48	[text3]	Health research may involve data collection in two ways: direct or indirect. Direct collection occurs when there is interaction with the participant, such as interviews, questionnaires, focus groups, in-person observations, blood collection, physical exams, clinical tests, or any other type of contact to obtain data or biological material. Indirect collection occurs when previously collected data, documents, images, or biological materials are used, without direct contact with the participant, even if they can be identified. This includes analysis of medical records, access to databases, previously performed exams, use of stored biological material, as well as images, recordings, or public/private information that enables identification of the participant.	Show field ONLY if: [qs_trabalha_campo] = 1.<br>Field type: descriptive
49	[qs_forma_coleta]	Which form of data collection have you worked with, directly or indirectly?	Show field ONLY if: [qs_trabalha_campo] = 1.<br>Field type: radio. Required. Options:<br>1: Direct<br>2: Indirect<br>3: Direct and Indirect<br>Custom alignment: RH
50	[text5]	Section header: FIELD RESEARCH CHALLENGES	Field type: descriptive

51	[qs_ha_desafios]	Do you consider that the field health research project has challenges?	Show field ONLY if: [ie_projetos_yn] = 1 AND [ie_idade_yn] = 1.<br>Field type: radio. Required. Options:<br>1: Yes<br>0: No
52	[qs_ha_desafios_quais]	Which challenges in the field health research project do you consider the most relevant? (Select all that apply)	Show field ONLY if: [qs_ha_desafios] = 1.<br>Field type: checkbox. Required. Options:<br>1: Climatic/Meteorological<br>2: Logistical (local infrastructure, transportation, etc.)<br>3: Ethical (consent, confidentiality, impact on the community, etc.)<br>4: Data quality / information collection<br>99: Other
53	[qs_ha_desafios_quais_outro]	Other (specify):	Show field ONLY if: [qs_ha_desafios_quais(99)] = 1.<br>Field type: text. Required
54	[qs_ha_desafios_log]	Which logistical challenges have you faced/face when carrying out the field health research project? (Select all that apply)	Show field ONLY if: [qs_ha_desafios_quais(2)] = 1.<br>Field type: checkbox. Required. Options:<br>1: Access to remote locations<br>2: Team accommodation<br>3: Team food<br>4: Storage of source documents / essential documents<br>5: Ineffective communication with local teams<br>6: Difficulty obtaining permissions or authorizations<br>7: Unexpected issues with equipment or materials<br>8: Limited time to carry out activities<br>9: Logistical costs<br>10: Constant lack of electricity<br>11: Lack of health infrastructure<br>12: Lack of basic sanitation<br>13: Supply and inventory management<br>14: Security and operational risks<br>15: Transportation<br>16: Transport of biological samples<br>99: Other
55	[qs_ha_desafios_log_outro]	Other logistical challenge (specify):	Show field ONLY if: [qs_ha_desafios_log(99)] = 1.<br>Field type: text. Required
56	[qs_ha_desafios_etic]	Which ethical challenges have you faced/face when carrying out the field health research project? (Select all that apply)	Show field ONLY if: [qs_ha_desafios_quais(3)] = 1.<br>Field type: checkbox. Required. Options:<br>1: Social, cultural, and value differences.<br>2: Ensuring anonymity and confidentiality of participants.<br>3: Impact of the research on local communities.<br>4: Obtaining the Informed Consent Form (ICF) and/or Assent Form.<br>5: Institutional pressures or conflicts of interest.<br>6: Power relationship between researcher(s) and participants.<br>7: Use and sharing of collected data.<br>99: Other

57	[qs_ha_desafios_etic_outro]	Other challenge(s) in obtaining the ICF or Assent (specify):	Show field ONLY if: [qs_ha_desafios_etic(99)] = 1.<br>Field type: text. Required
58	[qs_ha_desafios_etic_quais]	Which challenges in obtaining the ICF or Assent (Select all that apply):	Show field ONLY if: [qs_ha_desafios_etic(4)] = 1.<br>Field type: checkbox. Required. Options:<br>1: Language barriers<br>2: Distrust<br>3: Participant misinformation<br>4: Difficulty understanding<br>5: Difficulty communicating the objectives and methods of the research<br>99: Other
59	[qs_ha_desafios_etic_qual_outro]	Other ethical challenge(s) (specify):	Show field ONLY if: [qs_ha_desafios_etic_quais(99)] = 1.<br>Field type: text. Required
60	[qs_ha_desafios_cult_soc]	How do you address cultural and social issues when conducting the field health research project? (Select two main approaches)	Show field ONLY if: [qs_ha_desafios_etic(1)] = 1.<br>Field type: checkbox. Required. Options:<br>1: I do not address them<br>2: I adapt informational materials to the local culture<br>3: I consult local leaders to review the best language to be used for the target population<br>4: I integrate local residents into the research team<br>5: I prepare the community with educational actions that promote critical thinking and awareness about the research procedures and risks, enabling an informed decision about participation<br>99: Other<br>Annotations: @NONEOFTHEABOVE = 1
61	[qs_ha_desafios_cult_soc_outro]	Other cultural and social issues (specify):	Show field ONLY if: [qs_ha_desafios_cult_soc(99)] = 1.<br>Field type: text. Required
62	[text6]	Section header: ETHICS AND QUALITY. The following questions refer to quality and ethics/participant safety. Please answer considering exclusively the field health research project in which you work, belonging to the portfolio of the VPPCB/Fiocruz Clinical Research Platform.	Show field ONLY if: [ie_projetos_yn] = 1 AND [ie_idade_yn] = 1.<br>Field type: descriptive
63	[qs_info_riscos_beneficios]	Do you believe that participants recruited in the field health research project are/were sufficiently informed about the risks and benefits of the study?	Show field ONLY if: [ie_projetos_yn] = 1 AND [ie_idade_yn] = 1.<br>Field type: radio. Required. Options:<br>1: Yes<br>0: No<br>Custom alignment: RH
64	[qs_tcle_termo_assentimento]	In the field health research project, was an Informed Consent Form (ICF) and/or Assent Form obtained?	Show field ONLY if: [ie_projetos_yn] = 1 AND [ie_idade_yn] = 1.<br>Field type: radio. Required. Options:<br>1: Yes<br>0: No<br>Custom alignment: RH

65	[qs_registro_tcle]	Do you record (or did you record) the consent process in the project source document? (Assent form and/or ICF)	Show field ONLY if: [qs_tcle_termo_assentimento] = 1.<br>Field type: radio. Required. Options:<br>1: Yes<br>0: No<br>Custom alignment: RH
66	[qs_forma_registro_tcle]	How do (or did) you record the consent process?	Show field ONLY if: [qs_registro_tcle] = 1.<br>Field type: checkbox. Required. Options:<br>1: Description in the institution's medical record progress notes<br>2: Pre-filled form<br>3: Checklist<br>4: File note or similar<br>99: Other
67	[qs_forma_registro_tcle_outro]	Other (specify):	Show field ONLY if: [qs_forma_registro_tcle(99)] = 1.<br>Field type: text. Required
68	[text7]	According to Law No. 14,874 of May 28, 2024, which regulates research with human beings and establishes the National System of Ethics in Research with Human Beings, an Adverse Event (AE) is any unfavorable medical occurrence, whether or not related to the product under investigation, in a patient or research participant during the conduct of the study.	Show field ONLY if: [ie_projetos_yn] = 1 AND [ie_idade_yn] = 1.<br>Field type: descriptive
69	[qs_reporta_evento_adverso]	Do you collect (or did you collect) and report Adverse Events occurring with participants in the field health research project?	Show field ONLY if: [ie_projetos_yn] = 1 AND [ie_idade_yn] = 1.<br>Field type: radio. Required. Options:<br>1: Yes<br>0: No<br>96: Not applicable to the project<br>Custom alignment: RH
70	[text8]	According to Law No. 14,874 of May 28, 2024, which regulates research with human beings and establishes the National System of Ethics in Research with Human Beings, a Serious Adverse Event (SAE) is any unfavorable medical occurrence that results in: death, life-threatening risk, situations requiring hospitalization or prolongation of hospitalization, significant disability, congenital anomaly, or a clinically significant event.	Show field ONLY if: [ie_projetos_yn] = 1 AND [ie_idade_yn] = 1.<br>Field type: descriptive
71	[qs_reporta_evento_grave]	In case of a Serious Adverse Event (SAE) in the field health research project, do you collect the SAE and report it to the Sponsor and to the Research Ethics Committee (CEP)?	Show field ONLY if: [ie_projetos_yn] = 1 AND [ie_idade_yn] = 1.<br>Field type: radio. Required. Options:<br>1: Yes<br>0: No<br>96: Not applicable to the project<br>Custom alignment: RH

72	[text9]	Medical resources necessary to conduct field health research are the means, equipment, and structures that ensure the safety and well-being of participants and the research team. They are essential to prevent risks and to enable care in emergencies. These resources vary according to the type and location of the research and include: first-aid kit, PPE, an appropriate place for disposal of biological waste, an emergency action plan, prior knowledge of nearby health units, and emergency communication means in remote areas.	Show field ONLY if: [ie_projetos_yn] = 1 AND [ie_idade_yn] = 1.<br>Field type: descriptive
73	[qs_acesso_recursos_medicos]	Do you consider that you have/had adequate access to the medical resources necessary to carry out the field health research project?	Show field ONLY if: [ie_projetos_yn] = 1 AND [ie_idade_yn] = 1.<br>Field type: radio. Required. Options:<br>1: Yes<br>0: No
74	[qs_pop_existentes]	Does your research center have Standard Operating Procedures (SOPs) for executing the field health research project? (Select all that apply)	Show field ONLY if: [ie_projetos_yn] = 1 AND [ie_idade_yn] = 1.<br>Field type: checkbox. Required. Options:<br>1: Contingency Plan<br>2: CEP/Conep system process<br>3: Disposal of biological and non-biological materials<br>4: Team training and retraining<br>5: Recruitment and selection of research participants<br>6: Consent process and ICF obtaining<br>7: Source documents and CRFs verification<br>8: Use and calibration of equipment/instruments<br>9: Collection, transport, preparation, identification and analysis of laboratory samples<br>10: Receipt, accountability, destruction or return of investigational product<br>0: None of the above<br>Annotations: @NONEOFTHEABOVE = 0
75	[qs_pos_proced_exclus]	Are there Standard Operating Procedures (SOPs) established exclusively for the procedures involved in executing the field health research project?	Show field ONLY if: [ie_projetos_yn] = 1 AND [ie_idade_yn] = 1.<br>Field type: radio. Required. Options:<br>1: Yes<br>0: No<br>96: Not applicable to the project<br>Custom alignment: RH
76	[qs_pos_proced_exclus_desc]	List the main SOPs:	Show field ONLY if: [qs_pos_proced_exclus] = 1.<br>Field type: notes. Required

77	[qs_garantia_qualidade]	How do you ensure the quality of data collected during field health research? (Select two main actions)	Show field ONLY if: [ie_projetos_yn] = 1 AND [ie_idade_yn] = 1.<br>Field type: checkbox. Required. Options:<br>1: I do not ensure quality<br>2: Team training<br>3: Double-checking completed source documents<br>4: Review of clinical forms<br>5: Continuous supervision to ensure standardization and consistency of information<br>6: I use validated instruments<br>7: I use a validated electronic clinical record system (e.g., OpenClinica®, REDCap® etc.)<br>99: Other<br>Annotations: @NONEOFTHEABOVE = 1
78	[qs_garantia_qualidade_outro]	Other quality assurance action:	Show field ONLY if:<br>[qs_garantia_qualidade(99)] = 1.<br>Field type: text. Required
79	[qs_protocolo_pesquisa]	Do you follow a pre-established research protocol or research plan when conducting your field work?	Show field ONLY if: [ie_projetos_yn] = 1 AND [ie_idade_yn] = 1.<br>Field type: radio. Required. Options:<br>1: Yes, I always follow a formal protocol or plan<br>2: Sometimes, I follow a protocol but with flexibility<br>3: No, I do not follow a formal protocol<br>9: I am not sure
80	[text10]	Section header: FIELD RESEARCH OPPORTUNITIES. The questions below refer to opportunities in field research projects. Please answer considering exclusively the field health research project in which you work, belonging to the portfolio of the VPPCB/Fiocruz Clinical Research Platform.	Field type: descriptive
81	[qs_oportunidades_identificadas]	In your assessment, what are the main opportunities identified within the field health research project? (Select all that apply)	Show field ONLY if: [ie_projetos_yn] = 1 AND [ie_idade_yn] = 1.<br>Field type: checkbox. Required. Options:<br>1: Access to a specific population<br>2: Adaptation to local conditions (cultural, social, economic)<br>3: More representative data collection<br>4: Direct impact on the community<br>5: Institutional incentives<br>6: Improvement in public health policies<br>7: Learning opportunities with the local population<br>99: Other
82	[qs_oportunidades_identificadas_outro]	Other (specify):	Show field ONLY if:<br>[qs_oportunidades_identificadas(99)] = 1.<br>Field type: text. Required

83	[qs_impacto_comun]	In your perspective, what is the potential impact of the field health research project on the local community? (Select all that apply)	Show field ONLY if: [ie_projetos_yn] = 1 AND [ie_idade_yn] = 1.<br>Field type: checkbox. Required. Options:<br>1: Access to innovative treatments<br>2: Education about diseases<br>3: Engage communities in decision-making about health needs and treatments<br>4: Improved population health<br>5: No impact<br>99: Other<br>Annotations: @NONEOFTHEABOVE = 5
84	[qs_impacto_comun_outro]	Other impact (specify):	Show field ONLY if: [qs_impacto_comun(99)] = 1.<br>Field type: text. Required
85	[qs_conf_result]	Do you believe that participants in the field health research project show confidence in the results generated by the research?	Show field ONLY if: [ie_projetos_yn] = 1 AND [ie_idade_yn] = 1.<br>Field type: radio. Required. Options:<br>1: Yes, always<br>2: Sometimes<br>3: No<br>4: I am not sure
86	[qs_divulg_result]	How do you handle dissemination of the field health research results to participants?	Show field ONLY if: [ie_projetos_yn] = 1 AND [ie_idade_yn] = 1.<br>Field type: radio. Required. Options:<br>0: I do not disseminate<br>2: By distributing accessible reports (e-mail, social networks, etc.)<br>3: By offering direct feedback<br>4: By holding community meetings<br>99: Other
87	[qs_divulg_result_outro]	Other (specify):	Show field ONLY if: [qs_divulg_result] = 99.<br>Field type: text. Required
88	[text11]	Section header: CLINICAL RESEARCH. For the questions below, responses should be elaborated based exclusively on your professional experience.	Show field ONLY if: [ie_projetos_yn] = 1 AND [ie_idade_yn] = 1.<br>Field type: descriptive
89	[qs_pesquisa_campo_clinica]	Do you consider field health research to be clinical research?	Show field ONLY if: [ie_projetos_yn] = 1 AND [ie_idade_yn] = 1.<br>Field type: radio. Required. Options:<br>1: Yes<br>0: No<br>2: I am not sure
90	[qs_formacao_pesq_clin]	Have you ever participated in any course or training in clinical research? (Select all that apply)	Show field ONLY if: [ie_projetos_yn] = 1 AND [ie_idade_yn] = 1.<br>Field type: checkbox. Required. Options:<br>1: Long-term course (e.g., master's, PhD, lato sensu postgraduate program)<br>2: Short course (e.g., workshops, seminars)<br>3: Formal (courses, certifications, etc.)<br>4: Informal (mentoring, hands-on guidance)<br>5: Informal (during the Clinical Research Project Initiation Monitoring Visit)<br>0: I have never participated<br>Annotations: @NONEOFTHEABOVE = 0

91	[qs_visita_monitoria_fiocruz]	Was the Clinical Research Project Initiation Monitoring Visit conducted by the VPPCB/Fiocruz Clinical Research Platform team?	Show field ONLY if: [qs_formacao_pesq_clin(5)] = 1.<br>Field type: radio. Required. Options:<br>1: Yes<br>0: No<br>Custom alignment: RH
92	[qs_falta_formacao_influencia]	Do you believe that the lack of training in clinical research influences understanding of what characterizes clinical research?	Show field ONLY if: [ie_projetos_yn] = 1 AND [ie_idade_yn] = 1.<br>Field type: radio. Required. Options:<br>1: Yes, it directly influences<br>0: No, it does not influence<br>2: I am not sure
93	[qs_cultura_institucional]	Do you believe that your institution’s culture helps or hinders understanding of clinical research?	Show field ONLY if: [ie_projetos_yn] = 1 AND [ie_idade_yn] = 1.<br>Field type: radio. Required. Options:<br>1: Helps, because there is a strong emphasis on defining and practicing clinical research<br>0: Hinders, because there is no clear differentiation between clinical research and other types of research<br>2: I am not sure<br>95: Prefer not to answer
94	[questionario_eletronico_complete]	Form Status – Complete?	Field type: dropdown. Options:<br>0: Incomplete<br>1: Unverified<br>2: Complete
