# Supplementary material for: Challenges and opportunities in clinical field research: A Bourdieusian analysis of Fiocruz’s experience in Brazil
Source: PLoS One. 2026 Sep 25;21(9):e0359217. doi: 10.1371/journal.pone.0359217 (PMC13614590; doi:10.1371/journal.pone.0359217)
Supplement: Supplementary file 4 [file pone.0359217.s008.pdf]

September 04, 2026

Re: Resubmission of manuscript PONE-D-26-21934

Title: Challenges and opportunities in clinical field research: A Bourdieusian analysis of Fiocruz's experience in Brazil

Dear Dr. Rachel Suet Kay Chan and Reviewers,

We thank the Academic Editor and Reviewer #2 for their thorough evaluation of our manuscript and for acknowledging the substantial improvements made during the previous revision. We value the constructive feedback, which enabled us to further enhance the accuracy, reflexivity, and transparency of our work.

Below, we present a point-by-point response detailing how each comment was addressed in the revised manuscript. All corresponding revisions have been highlighted in the "Revised Manuscript with Tracked Changes" file and incorporated into the "Manuscript" file (clean version).

Responses to Reviewer #2

Comment 1: A residual artifact of the skip-logic design remains in the prose, even though it was corrected in the tables. The Discussion states: "The statistical evidence is compelling: 100% of researchers who identified misinformation, mistrust, or language barriers also perceived a general challenge in the consent process." Given the questionnaire's branching logic, participants were only asked about specific consent barriers if they had already indicated a general consent difficulty — so 100% overlap is a structural property of the instrument, not an empirical finding. I would ask the authors to either remove this framing or explicitly acknowledge it reflects the skip-logic design rather than an independent result.

Response: We thank the reviewer for highlighting this oversight. We recognize that this 100% overlap was an artifact of the questionnaire's branching logic rather than an independent empirical finding. Accordingly, we have removed this inappropriate statistical framing from the Discussion. The revised text now presents these findings descriptively within the subgroup of participants who encountered difficulties during the consent process, without making unwarranted inferential claims.

- Location in text: Discussion [Page 25, Lines 556-560]): "…Among researchers who reported difficulties in the consent process, barriers such as participants' comprehension challenges, misinformation, and mistrust were particularly prominent."

Comment 2: Claims are still generalized beyond the study setting in places that were not addressed in the previous revision. The Introduction and Discussion make several statements extending the findings to "LMICs," "the Global South," and "developing health systems" broadly (e.g., describing Fiocruz's experience as "a replicable model for other middle-income nations"). The authors have appropriately added hedging language regarding the sample size and representativeness elsewhere in the manuscript, but this same caution has not been extended to these broader geopolitical claims, which remain based solely on a convenience sample from six Fiocruz-funded projects in Brazil. I'd recommend qualifying these statements or explicitly noting them as speculative extrapolation.

Response: We thank the reviewer for this critical observation. We fully agree that drawing broad, systemic conclusions regarding "low- and middle-income countries" (LMICs), the "Global South," or "developing health systems" based on an exploratory convenience sample (n = 51)—comprising six research projects funded by Fiocruz—goes beyond the study's empirical basis. In response, we have revised the Introduction, Discussion, and Conclusion to eliminate sweeping generalizations, remove prescriptive statements regarding transferability, and explicitly qualify comparisons across different contexts as situated, exploratory reflections. Below, we present the key revisions made to align the text with the study's scope:

- Location in text: Introduction [Page 3, Lines 73-78]): "Given this extensive reach and expertise, Fiocruz's institutional experience across a geographically heterogeneous territory provides valuable contextual insights that can inform broader discussions on clinical research governance in resource-limited settings, while acknowledging that direct transferability is inherently constrained by local systemic specificities."
- Location in text: Introduction [Page 5, Lines 101-103]): "These obstacles, while observed within specific Brazilian research settings, illustrate operational and logistical challenges that may resonate across developing health systems..."
- Location in text: Introduction [Page 5, Lines 112-115]): "By addressing this need, this study provides a situated diagnosis of this institutional setting and offers an exploratory contribution to debates surrounding the operational sustainability of clinical research in resource-limited settings."

- Location in text: Conclusion [Page 29, Lines 671-673]): “This multiplicity of perspectives can strengthen the design of clinical research, favoring approaches that are more sensitive and responsive to the operational and social realities of vulnerable territories in Brazil and potentially comparable settings.”

Comment 3: The external comparator used to support the “academic whiteness” argument is not well matched to this study. The Discussion cites data from a different institution (UFMT) on racial composition of principal investigators to support claims of structural racial asymmetry within this study's sample (which measured all research team members, not just PIs). I would ask the authors to either better justify the comparability of these two data sources or soften the claim.

Response: We thank the reviewer for this critical observation. We agree that our sample—which includes team members occupying diverse technical, operational, and research roles—cannot be directly equated to data restricted exclusively to principal investigators (project coordinators) at UFMT.

In response, we have:

1. Retitled the subsection to a more objective and analytically precise heading: “The geopolitics of scientific capital and demographic asymmetries”;
2. Softened the claim by explicitly noting that our sample comprised diverse operational and research roles rather than solely PIs;
3. Clarified that the UFMT institutional data are introduced solely as a broader contextual reference illustrating leadership disparity in the national academic field, rather than as a direct methodological counterpart;
4. Emphasized that direct equivalencies must be interpreted with caution.

- Location in text: Discussion [Page 20, Lines 442-443]): “The geopolitics of scientific capital and demographic asymmetries.”
- Location in text: Discussion [Page 21, Lines 457-461]): “While our sample encompassed research team members across diverse operational and technical roles rather than exclusively principal investigators, the demographic profile observed (68.6% white) resonates with broader structural asymmetries documented in the Brazilian scientific field. For contextual comparison, institutional data on research project leadership…”
- Location in text: Discussion [Page 21, Lines 464-466]): “Although direct equivalencies between general field team composition and principal investigator demographics must be drawn with caution, both indicators collectively illustrate...”

- Location in text: Discussion [Page 21, Lines 468 - 471]): “The persistence of these geographic and racial concentrations may hinder the incorporation of perspectives aimed at methodological and theoretical diversification, which are essential for fostering plural and inclusive knowledge in public health.”

Comment 4: Out of the 67 participants recruited, 51 comprised the final sample; a brief breakdown of the 16 exclusions would improve transparency, though this is not essential.

Response: We appreciate this suggestion and have incorporated the complete breakdown of the 16 exclusions into the Results section under "Participant profile" to enhance transparency and reporting standards.

Location in text: Results [Page 11, Lines 250 - 253]): “The 16 exclusions comprised: participants who signed the ICF but did not complete the questionnaire (n = 7), duplicate questionnaire entries (n = 3), and failure to meet the eligibility criterion of having at least one year of clinical field research experience (n = 6).”

We believe the manuscript has been greatly improved by incorporating these revisions. We are deeply grateful to the editors and reviewers for their guidance.

We look forward to hearing from you regarding the status of our submission.

Sincerely,

Renata Caldeira Diniz

On behalf of all co-authors

Oswaldo Cruz Foundation (Fiocruz) / Hospital Alemão Oswaldo Cruz

Email: rdiniz2003@gmail.com

Phone: +55 (31) 99116-6047
