# Supplementary material for: Challenges and opportunities in clinical field research: A Bourdieusian analysis of Fiocruz’s experience in Brazil
Source: PLoS One. 2026 Sep 25;21(9):e0359217. doi: 10.1371/journal.pone.0359217 (PMC13614590; doi:10.1371/journal.pone.0359217)
Supplement: Supplementary file 5 [file pone.0359217.s007.docx]

**July 11, 2026**

**Re:** Resubmission of manuscript PONE-D-26-21934

**Title:** Challenges and opportunities in clinical field research: A Bourdieusian analysis of Fiocruz’s experience in Brazil

**Dear Dr. Rachel Suet Kay Chan and Reviewers**,

We would like to thank the Academic Editor and the Reviewers for their time, constructive feedback, and insightful comments. We have carefully considered all the points raised and have significantly revised the manuscript to address them.

Below, we provide a point-by-point response detailing how and where each comment was addressed in the revised manuscript, with references to the respective pages and lines in the revised file.

**PART I: Response to Journal Requirements**

**Requirement 1:** *Ensure that your manuscript meets PLOS ONE's style requirements, including those for file naming.*

**Response:** We have reviewed the manuscript and confirm that it complies with PLOS ONE’s style and formatting requirements.

**Requirement 2:** *The ethical approval number(s) listed in the manuscript and/or submission metadata does not match the approval number on the ethical approval document you provided. Please ensure that all approval numbers are correct.*

**Response:** We apologize for this oversight. We have corrected the inconsistency. The ethical approval number now correctly reads Opinion No. 7.698.450 in both the “Ethics statement” section and the “S1 Appendix” description.

- Location in text (Materials and methods > Ethics statement, [Lines 122]): “The study was approved by the Research Ethics Committee of the Oswaldo Cruz Institute (CAAE: 89038525.6.0000.5248; Opinion No. 7.698.450) (S1 Appendix).”
- Location in text (Supporting information, [Lines 735]): “S1 Appendix. Ethical Approval Document. Copy of the consolidated opinion from the Research Ethics Committee of the Oswaldo Cruz Institute (Opinion No. 7.698.450).”

**Requirement 3 & 4:** *Amend Financial Disclosure Statement to include all funding sources and the exact phrase: “There was no additional external funding received for this study.”*

**Response:** We have updated the Financial Disclosure Statement in the online submission system and added it directly to the end of the manuscript as requested.

- This study was funded by the Social Responsibility department of Hospital Alemão Oswaldo Cruz through the Institutional Development Support Program of the Unified Health System (PROADI-SUS), as part of the Specialization Course in Clinical Research. The support was awarded to RCD (covering the three-year term 2023-2026). The funder had no role in study design, data collection and analysis, decision to publish, or preparation of the manuscript. *There was no additional external funding received for this study.*

**Requirement 5:** *Amend Competing Interests to state exactly: “The authors have declared that no competing interests exist.”*

**Response:** We have updated the Competing Interests statement in the online system and added it to the end of the manuscript.

- “The authors have declared that no competing interests exist.”

**PART II: Response to Reviewer #1**

**Reviewer #1, Point 1:** *Major concern about the statistical test and identification of significance in Table 2, 3, 4. The questionnaire is descriptive and skip-logic design... Therefore, the statistical difference between yes and no is largely uninformative - it might have been better if yes was sub divided into how strong the challenge had been.*

**Response:** We completely agree with the reviewer's methodological concern regarding the skip-logic design. Testing for statistical significance between “yes” and “no” groups was inappropriate for these specific sub-variables. We have entirely removed all inferential statistical tests and p-values from the manuscript. The quantitative analysis is now strictly descriptive, and Tables 2, 3, and 4 now correctly display only the absolute and relative frequencies (percentages) of the observed barriers.

- Location in text (Materials and methods > Data Analysis, [Page 9, Lines 217-218]): “Quantitative data were analyzed using descriptive statistics (absolute and relative frequencies)...” *(Note: Tables 2, 3, and 4 have also been updated accordingly).*

**Reviewer #1, Point 2:** *Could the authors clarify the choice of selection of Bourdieu framework? What was the primary objective for choosing this, how was it utilized and what results implied through that. That's not sufficiently described in manuscript.*

**Response:** Thank you for highlighting this gap. We have significantly expanded the Introduction to better justify the choice of Bourdieusian sociology as our theoretical lens. We clarified that operational challenges in LMICs reflect deeper, often invisible structures of power, prestige, and resource allocation that traditional epidemiological frameworks fail to capture.

- Location in text (Introduction, [Page 5, Lines 113-117]): “The choice of Bourdieusian sociology is justified because operational challenges, particularly in low- and middle-income countries, are rarely merely technical. They are direct reflections of asymmetries in power, prestige, and resource allocation—often invisible structures that underpin the scientific field...”

**Reviewer #1, Point 3:** *Could authors elaborate on particular logistical obstacle, access to remote locations? How did authors utilize Bourdieusian here?*

**Response:** We have expanded the discussion on logistical challenges to integrate Bourdieusian concepts, specifically explaining how geography acts as a structural barrier that dictates the accumulation of capital.

- Location in text (Discussion > Structural barriers... [Page 22, Lines 481-483; 492-494): “The high prevalence of logistical obstacles, particularly regarding access to remote locations, reveals that geography acts as a structural barrier that constrains knowledge production and health equity... This fact reveals that the symbolic capital and institutional prestige of entities such as Fiocruz do not always translate into the economic capital necessary to ensure the operational safety of teams.”

**Reviewer #1, Point 4:** *The overall responses are fewer and some are based on 1/2 responses. The authors are advised to exercise caution and avoid over-interpretation. With the results present, it will likely be hypothesis generating.*

**Response:** We appreciate this important point. We have adjusted the tone throughout the manuscript to avoid over-interpretation. We explicitly acknowledge the sample size limitations and have framed the study as hypothesis-generating.

- Location in text (Materials and methods > Study population and sample, [Page 8, Lines 187-189]): “Due to the relatively small sample size, the findings are largely considered hypothesis-generating, establishing a baseline for broader future investigations.”

**Reviewer #1, Point 5:** *The authors describe in limitations that community perspectives were absent. However, they would form a strong pillar to this study and not a trivial limitation.*

**Response:** We strongly agree. We have rewritten the limitations paragraph in the Conclusion to emphasize that the absence of community voices is a critical empirical gap.

- Location in text (Conclusion, [Page 28, Lines 646-649]): “The design relied exclusively on the perceptions of researchers—agents of the scientific field—which prevents a complete triangulation of power relations due to the absence of voices from institutional managers and, crucially, from local community participants. Without the community’s perspective, the trust perceived by the researchers’ risks being a projection of their own academic habitus, failing to detect subtle forms of resistance or symbolic violence.”

**PART III: Response to Reviewer #2**

**Reviewer #2, Point 1 & 5:** *1. The rationale for several statistical analyses requires further clarification... 5. Given the number of statistical tests performed, the authors should discuss whether any consideration was given to multiple comparisons and the potential risk of false-positive findings.*

**Response:** We agree with these methodological concerns. As addressed in our response to Reviewer #1, we have removed all inferential statistical analyses (p-values) from the manuscript. By transitioning to a purely descriptive approach (frequencies and percentages), we have eliminated the issues regarding uninterpretable associations and the risk of false-positives from multiple comparisons.

**Reviewer #2, Point 2:** *The qualitative component appears to be based on open-ended responses from a relatively small subset of participants. While the findings are interesting, some of the broader theoretical interpretations regarding symbolic violence... appear stronger than the available qualitative evidence may support. The distinction between empirical findings and theoretical interpretation should be made more explicit.*

**Response:** We thank the reviewer for this constructive criticism. We have clarified the sample size of the qualitative subset (n = 13) in the methodology and tempered our theoretical claims in the Discussion and Conclusion, framing them as theoretical inferences rather than absolute empirical facts.

- Location in text (Conclusion, [Page 28, Lines 643-646]): “It is recognized that qualitative data collection through “other” open-ended fields and voluntary participation may have selected participants with more critical views, potentially limiting the capture of deeper subjectivities.”

**Reviewer #2, Point 3:** *The discussion includes extensive consideration of community trust... Since community members were not directly surveyed, interpretations regarding community trust should be presented more cautiously and framed as researchers' perceptions rather than direct evidence of community views.*

**Response:** We fully agree. We have revised the text and figures to ensure that “community trust” is strictly framed as the researchers' *perception* of that trust.

- Location in text (Results > Fig 4 title, [Page 19, lines 429]): “Fig 4. Researchers’ perception of community trust in research findings.”
- Location in text (Discussion > The paradox of engagement..., [Page 26, Lines 586-589; 591-594]): “...there is a predominant belief that the community “always” (60.8%) or “sometimes” (29.4%) trusts the research findings... this unilateral perception may mask subtle dynamics of symbolic violence, in which the silence or absence of questioning on the part of the community ends up being interpreted by the research teams as full acceptance...”

**Reviewer #2, Point 4:** *The manuscript would benefit from additional methodological detail regarding the qualitative analysis, including coding procedures, coder involvement and steps taken to ensure analytic rigor and consistency.*

**Response:** We have expanded the "Data Analysis" section to provide a detailed description of our qualitative methodology, including the software used, coding structure, and peer validation process.

- Location in text (Methods > Data analysis, [Page 9. Lines 219-221; Page 10, 223-225]): “Exclusively for the analysis of qualitative data derived from open-ended responses and optional “other” fields, completed by 13 participants, the MAXQDA 26 software was employed, following a hierarchical thematic coding protocol... coding was conducted independently by two of the authors and subjected to peer validation to resolve any discrepancies through consensus."

We believe the manuscript has been greatly improved by incorporating these revisions. We are deeply grateful to the editors and reviewers for their guidance.

We look forward to hearing from you regarding the status of our submission.

Sincerely,

**Renata Caldeira Diniz**

On behalf of all co-authors

Oswaldo Cruz Foundation (Fiocruz) / Hospital Alemão Oswaldo Cruz

Email: [rdiniz2003@gmail.com](mailto:rdiniz2003@gmail.com)

Phone: +55 (31) 99116-6047
