# Supplementary material for: Challenges and opportunities in clinical field research: A Bourdieusian analysis of Fiocruz’s experience in Brazil
Source: PLoS One. 2026 Sep 25;21(9):e0359217. doi: 10.1371/journal.pone.0359217 (PMC13614590; doi:10.1371/journal.pone.0359217)
Supplement: Peer review history

# Peer review: Decision Letter 0

**DOI:** 10.1371/journal.pone.0359217.r001

**Authors:** Rachel Suet Kay Chan

1 Jun 2026

Dear Dr. Diniz,

Thank you for submitting your manuscript to PLOS ONE. After careful consideration, we feel that it has merit but does not fully meet PLOS ONE’s publication criteria as it currently stands. Therefore, we invite you to submit a revised version of the manuscript that addresses the points raised during the review process.

==============================

Dear Author/s

The reviewers have recommended a Major Revision.

Their comments are below.

Best regards

==============================

Please submit your revised manuscript by Jul 31 2026 11:59PM. If you will need more time than this to complete your revisions, please reply to this message or contact the journal office at plosone@plos.org. When you're ready to submit your revision, log on to https://www.editorialmanager.com/pone/ and select the 'Submissions Needing Revision' folder to locate your manuscript file.

- A letter that responds to each point raised by the academic editor and reviewer(s). You should upload this letter as a separate file labeled 'Response to Reviewers'.
- A marked-up copy of your manuscript that highlights changes made to the original version. You should upload this as a separate file labeled 'Revised Manuscript with Track Changes'.
- An unmarked version of your revised paper without tracked changes. You should upload this as a separate file labeled 'Manuscript'.

If you would like to make changes to your financial disclosure, please include your updated statement in your cover letter. Guidelines for resubmitting your figure files are available below the reviewer comments at the end of this letter.

If applicable, we recommend that you deposit your laboratory protocols in protocols.io to enhance the reproducibility of your results. Protocols.io assigns your protocol its own identifier (DOI) so that it can be cited independently in the future. For instructions see: https://journals.plos.org/plosone/s/submission-guidelines#loc-laboratory-protocols. Additionally, PLOS ONE offers an option for publishing peer-reviewed Lab Protocol articles, which describe protocols hosted on protocols.io. Read more information on sharing protocols at https://plos.org/protocols?utm_medium=editorial-email&utm_source=authorletters&utm_campaign=protocols.

As the corresponding author, your ORCID iD is verified in the submission system and will appear in the published article. PLOS supports the use of ORCID, and we encourage all coauthors to register for an ORCID iD and use it as well. Please encourage your coauthors to verify their ORCID iD within the submission system before final acceptance, as unverified ORCID iDs will not appear in the published article. *Only*  the individual author can complete the verification step; PLOS staff *cannot*  verify ORCID iDs on behalf of authors.

We look forward to receiving your revised manuscript.

Kind regards,

Rachel Suet Kay Chan, Ph.D.

Academic Editor

PLOS One

Journal Requirements:

When submitting your revision, we need you to address these additional requirements.

1. Please ensure that your manuscript meets PLOS ONE's style requirements, including those for file naming. The PLOS ONE style templates can be found at

https://journals.plos.org/plosone/s/file?id=wjVg/PLOSOne_formatting_sample_main_body.pdf and

https://journals.plos.org/plosone/s/file?id=ba62/PLOSOne_formatting_sample_title_authors_affiliations.pdf

2. The ethical approval number(s) listed in the manuscript and/or submission metadata does not match the approval number on the ethical approval document you provided. Please ensure that all approval numbers are correct.

3. We note that the grant information you provided in the ‘Funding Information’ and ‘Financial Disclosure’ sections do not match.

When you resubmit, please ensure that you provide the correct grant numbers for the awards you received for your study in the ‘Funding Information’ section.

4. Thank you for stating in your Funding Statement:

“Financial Disclosure Statement

This study was conducted in collaboration with the Social Responsibility department of Hospital Alemão Oswaldo Cruz (https://www.hospitaloswaldocruz.org.br/) and supported by the Brazilian Ministry of Health (https://www.gov.br/saude/pt-br) through the Institutional Development Support Program of the Unified Health System (PROADI-SUS), as part of the Specialization Course in Clinical Research. RCD received support through this program. The funders had no role in study design, data collection and analysis, decision to publish, or preparation of the manuscript.”

Please provide an amended statement that declares *all* the funding or sources of support (whether external or internal to your organization) received during this study, as detailed online in our guide for authors at http://journals.plos.org/plosone/s/submit-now. Please also include the statement “There was no additional external funding received for this study.” in your updated Funding Statement.

Please include your amended Funding Statement within your cover letter. We will change the online submission form on your behalf.

5. Thank you for stating the following in your Competing Interests section:

“The authors declared that there are no conflicts of interest.”

Please complete your Competing Interests on the online submission form to state any Competing Interests. If you have no competing interests, please state "The authors have declared that no competing interests exist.", as detailed online in our guide for authors at http://journals.plos.org/plosone/s/submit-now

This information should be included in your cover letter; we will change the online submission form on your behalf.

6. If the reviewer comments include a recommendation to cite specific previously published works, please review and evaluate these publications to determine whether they are relevant and should be cited. There is no requirement to cite these works unless the editor has indicated otherwise.

Additional Editor Comments:

Dear Author/s

The reviewers have recommended a Major Revision.

Their comments are below.

Best regards

[Note: HTML markup is below. Please do not edit.]

Reviewers' comments:

Reviewer's Responses to Questions

**Comments to the Author**

1. Is the manuscript technically sound, and do the data support the conclusions?

Reviewer #1: Partly

Reviewer #2: Partly

**********

2. Has the statistical analysis been performed appropriately and rigorously?

Reviewer #1: No

Reviewer #2: Yes

**********

3. Have the authors made all data underlying the findings in their manuscript fully available?

The PLOS Data policy

Reviewer #1: Yes

Reviewer #2: Yes

**********

4. Is the manuscript presented in an intelligible fashion and written in standard English?

Reviewer #1: Yes

Reviewer #2: Yes

**********

Reviewer #1: Overall Assessment

The study aim is quite unexplored but a crucial area to be studied. This is relevant both clinically and for epidemiologists for good practice research. The authors examines the challenges on clinical field research and this informs good epidemiological practice and serve as a good tool for education. It's timely and a topic worthy to be studied.

The social determinants impact research greatly - this has been known but authors delve deeper into the operational challenges during field research in LMIC context. The choice of Bourdieusian lens is interesting.

I have some concerns -

1. Major concern about the statistical test and identification of signficance in Table 2, 3, 4. The questionnaire is descriptive and skip-logic design. The subtypes are filled by only those who confirmed having challenges. It's unclear why the testing is between "no" vs. "yes" group rather than degrees of yes? By design, did authors expect the "no" column to have any values? If challenge is no, patients wouldn't even fill the subtype of challenge. Therefore, the statistical difference between yes and no is largely uninformative - it might have been better if yes was sub divided into how strong the challenge had been.

2. Could the authors clarify the choice of selection of Bourdieu framework? What was the primary objective for choosing this, how was it utilized and what results implied through that. That's not sufficiently described in manuscript.

3. Could authors elaborate on particular logistical obstacle, access to remote locations? How did authors utilize Bourdieusian here?

4. The overall responses are fewer and some are based on 1/2 responses. The authors are advised to exercise caution and avoid over-interpretation. With the results present, it will likely be hypotheis generating.

5. The authors describe in limitations that community perspectives were absent. However, they would form a strong pillar to this study and not a trivial limitation.

Reviewer #2: 1. The rationale for several statistical analyses requires further clarification. In multiple instances, specific barriers appear to be tested against broader challenge categories that already encompass those barriers. This creates uncertainty regarding the interpretability and added value of some reported associations. The authors should provide a clearer explanation of the hypotheses being tested and justify the analytical strategy.

2. The qualitative component appears to be based on open-ended responses from a relatively small subset of participants. While the findings are interesting, some of the broader theoretical interpretations regarding symbolic violence, power asymmetries, social distance and scientific authority appear stronger than the available qualitative evidence may support. The distinction between empirical findings and theoretical interpretation should be made more explicit.

3. The discussion includes extensive consideration of community trust and researcher-community relationships. However, data were collected exclusively from researchers. Since community members were not directly surveyed, interpretations regarding community trust should be presented more cautiously and framed as researchers’ perceptions rather than direct evidence of community views.

4. The manuscript would benefit from additional methodological detail regarding the qualitative analysis, including coding procedures, coder involvement and steps taken to ensure analytic rigor and consistency.

5. Given the number of statistical tests performed, the authors should discuss whether any consideration was given to multiple comparisons and the potential risk of false-positive findings.

**********

what does this mean?). If published, this will include your full peer review and any attached files.

If you choose “no”, your identity will remain anonymous but your review may still be made public.

**Do you want your identity to be public for this peer review?**  For information about this choice, including consent withdrawal, please see our Privacy Policy

Reviewer #1: No

Reviewer #2: **Yes:** Keshav Banga

**********

[NOTE: If reviewer comments were submitted as an attachment file, they will be attached to this email and accessible via the submission site. Please log into your account, locate the manuscript record, and check for the action link "View Attachments". If this link does not appear, there are no attachment files.]

To ensure your figures meet our technical requirements, please review our figure guidelines: https://journals.plos.org/plosone/s/figures

You may also use PLOS’s free figure tool, NAAS, to help you prepare publication quality figures: https://journals.plos.org/plosone/s/figures#loc-tools-for-figure-preparation.

NAAS will assess whether your figures meet our technical requirements by comparing each figure against our figure specifications.

# Author comment: Author response to Decision Letter 1

**DOI:** 10.1371/journal.pone.0359217.r002

11 Jul 2026

PART I: Response to Journal Requirements

Requirement 1: Ensure that your manuscript meets PLOS ONE's style requirements, including those for file naming.

Response: We have reviewed the manuscript and confirm that it complies with PLOS ONE’s style and formatting requirements.

Requirement 2: The ethical approval number(s) listed in the manuscript and/or submission metadata does not match the approval number on the ethical approval document you provided. Please ensure that all approval numbers are correct.

Response: We apologize for this oversight. We have corrected the inconsistency. The ethical approval number now correctly reads Opinion No. 7.698.450 in both the “Ethics statement” section and the “S1 Appendix” description.

• Location in text (Materials and methods > Ethics statement, [Lines 122]): “The study was approved by the Research Ethics Committee of the Oswaldo Cruz Institute (CAAE: 89038525.6.0000.5248; Opinion No. 7.698.450) (S1 Appendix).”

• Location in text (Supporting information, [Lines 735]): “S1 Appendix. Ethical Approval Document. Copy of the consolidated opinion from the Research Ethics Committee of the Oswaldo Cruz Institute (Opinion No. 7.698.450).”

Requirement 3 & 4: Amend Financial Disclosure Statement to include all funding sources and the exact phrase: “There was no additional external funding received for this study.”

Response: We have updated the Financial Disclosure Statement in the online submission system and added it directly to the end of the manuscript as requested.

• This study was funded by the Social Responsibility department of Hospital Alemão Oswaldo Cruz through the Institutional Development Support Program of the Unified Health System (PROADI-SUS), as part of the Specialization Course in Clinical Research. The support was awarded to RCD (covering the three-year term 2023-2026). The funder had no role in study design, data collection and analysis, decision to publish, or preparation of the manuscript. There was no additional external funding received for this study.

Requirement 5: Amend Competing Interests to state exactly: “The authors have declared that no competing interests exist.”

Response: We have updated the Competing Interests statement in the online system and added it to the end of the manuscript.

• “The authors have declared that no competing interests exist.”

PART II: Response to Reviewer #1

Reviewer #1, Point 1: Major concern about the statistical test and identification of significance in Table 2, 3, 4. The questionnaire is descriptive and skip-logic design... Therefore, the statistical difference between yes and no is largely uninformative - it might have been better if yes was sub divided into how strong the challenge had been.

Response: We completely agree with the reviewer's methodological concern regarding the skip-logic design. Testing for statistical significance between “yes” and “no” groups was inappropriate for these specific sub-variables. We have entirely removed all inferential statistical tests and p-values from the manuscript. The quantitative analysis is now strictly descriptive, and Tables 2, 3, and 4 now correctly display only the absolute and relative frequencies (percentages) of the observed barriers.

• Location in text (Materials and methods > Data Analysis, [Page 9, Lines 217-218]): “Quantitative data were analyzed using descriptive statistics (absolute and relative frequencies)...” (Note: Tables 2, 3, and 4 have also been updated accordingly).

Reviewer #1, Point 2: Could the authors clarify the choice of selection of Bourdieu framework? What was the primary objective for choosing this, how was it utilized and what results implied through that. That's not sufficiently described in manuscript.

Response: Thank you for highlighting this gap. We have significantly expanded the Introduction to better justify the choice of Bourdieusian sociology as our theoretical lens. We clarified that operational challenges in LMICs reflect deeper, often invisible structures of power, prestige, and resource allocation that traditional epidemiological frameworks fail to capture.

• Location in text (Introduction, [Page 5, Lines 113-117]): “The choice of Bourdieusian sociology is justified because operational challenges, particularly in low- and middle-income countries, are rarely merely technical. They are direct reflections of asymmetries in power, prestige, and resource allocation—often invisible structures that underpin the scientific field...”

Reviewer #1, Point 3: Could authors elaborate on particular logistical obstacle, access to remote locations? How did authors utilize Bourdieusian here?

Response: We have expanded the discussion on logistical challenges to integrate Bourdieusian concepts, specifically explaining how geography acts as a structural barrier that dictates the accumulation of capital.

• Location in text (Discussion > Structural barriers... [Page 22, Lines 481-483; 492-494): “The high prevalence of logistical obstacles, particularly regarding access to remote locations, reveals that geography acts as a structural barrier that constrains knowledge production and health equity... This fact reveals that the symbolic capital and institutional prestige of entities such as Fiocruz do not always translate into the economic capital necessary to ensure the operational safety of teams.”

Reviewer #1, Point 4: The overall responses are fewer and some are based on 1/2 responses. The authors are advised to exercise caution and avoid over-interpretation. With the results present, it will likely be hypothesis generating.

Response: We appreciate this important point. We have adjusted the tone throughout the manuscript to avoid over-interpretation. We explicitly acknowledge the sample size limitations and have framed the study as hypothesis-generating.

• Location in text (Materials and methods > Study population and sample, [Page 8, Lines 187-189]): “Due to the relatively small sample size, the findings are largely considered hypothesis-generating, establishing a baseline for broader future investigations.”

Reviewer #1, Point 5: The authors describe in limitations that community perspectives were absent. However, they would form a strong pillar to this study and not a trivial limitation.

Response: We strongly agree. We have rewritten the limitations paragraph in the Conclusion to emphasize that the absence of community voices is a critical empirical gap.

• Location in text (Conclusion, [Page 28, Lines 646-649]): “The design relied exclusively on the perceptions of researchers—agents of the scientific field—which prevents a complete triangulation of power relations due to the absence of voices from institutional managers and, crucially, from local community participants. Without the community’s perspective, the trust perceived by the researchers’ risks being a projection of their own academic habitus, failing to detect subtle forms of resistance or symbolic violence.”

PART III: Response to Reviewer #2

Reviewer #2, Point 1 & 5: 1. The rationale for several statistical analyses requires further clarification... 5. Given the number of statistical tests performed, the authors should discuss whether any consideration was given to multiple comparisons and the potential risk of false-positive findings.

Response: We agree with these methodological concerns. As addressed in our response to Reviewer #1, we have removed all inferential statistical analyses (p-values) from the manuscript. By transitioning to a purely descriptive approach (frequencies and percentages), we have eliminated the issues regarding uninterpretable associations and the risk of false-positives from multiple comparisons.

Reviewer #2, Point 2: The qualitative component appears to be based on open-ended responses from a relatively small subset of participants. While the findings are interesting, some of the broader theoretical interpretations regarding symbolic violence... appear stronger than the available qualitative evidence may support. The distinction between empirical findings and theoretical interpretation should be made more explicit.

Response: We thank the reviewer for this constructive criticism. We have clarified the sample size of the qualitative subset (n = 13) in the methodology and tempered our theoretical claims in the Discussion and Conclusion, framing them as theoretical inferences rather than absolute empirical facts.

• Location in text (Conclusion, [Page 28, Lines 643-646]): “It is recognized that qualitative data collection through “other” open-ended fields and voluntary participation may have selected participants with more critical views, potentially limiting the capture of deeper subjectivities.”

Reviewer #2, Point 3: The discussion includes extensive consideration of community trust... Since community members were not directly surveyed, interpretations regarding community trust should be presented more cautiously and framed as researchers' perceptions rather than direct evidence of community views.

Response: We fully agree. We have revised the text and figures to ensure that “community trust” is strictly framed as the researchers' perception of that trust.

• Location in text (Results > Fig 4 title, [Page 19, lines 429]): “Fig 4. Researchers’ perception of community trust in research findings.”

• Location in text (Discussion > The paradox of engagement..., [Page 26, Lines 586-589; 591-594]): “...there is a predominant belief that the community “always” (60.8%) or “sometimes” (29.4%) trusts the research findings... this unilateral perception may mask subtle dynamics of symbolic violence, in which the silence or absence of questioning on the part of the community ends up being interpreted by the research teams as full acceptance...”

Reviewer #2, Point 4: The manuscript would benefit from additional methodological detail regarding the qualitative analysis, including coding procedures, coder involvement and steps taken to ensure analytic rigor and consistency.

Response: We have expanded the "Data Analysis" section to provide a detailed description of our qualitative methodology, including the software used, coding structure, and peer validation process.

• Location in text (Methods > Data analysis, [Page 9. Lines 219-221; Page 10, 223-225]): “Exclusively for the analysis of qualitative data derived from open-ended responses and optional “other” fields, completed by 13 participants, the MAXQDA 26 software was employed, following a hierarchical thematic coding protocol... coding was conducted independently by two of the authors and subjected to peer validation to resolve any discrepancies through consensus."

We believe the manuscript has been greatly improved by incorporating these revisions. We are deeply grateful to the editors and reviewers for their guidance.

We look forward to hearing from you regarding the status of our submission.

Sincerely,

Renata Caldeira Diniz

On behalf of all co-authors

Attachment Submitted filename: Response_to_Reviewers_PONE-D-26-21934.docx

# Peer review: Decision Letter 1

**DOI:** 10.1371/journal.pone.0359217.r003

**Authors:** Rachel Suet Kay Chan

4 Sep 2026

Dear Dr. Diniz,

Thank you for submitting your manuscript to PLOS One. After careful consideration, we feel that it has merit but does not fully meet PLOS One’s publication criteria as it currently stands. Therefore, we invite you to submit a revised version of the manuscript that addresses the points raised during the review process.

Please submit your revised manuscript by Oct 03 2026 11:59PM. If you will need more time than this to complete your revisions, please reply to this message or contact the journal office at plosone@plos.org. When you're ready to submit your revision, log on to https://www.editorialmanager.com/pone/ and select the 'Submissions Needing Revision' folder to locate your manuscript file.

- A letter that responds to each point raised by the academic editor and reviewer(s). You should upload this letter as a separate file labeled 'Response to Reviewers'.
- A marked-up copy of your manuscript that highlights changes made to the original version. You should upload this as a separate file labeled 'Revised Manuscript with Track Changes'.
- An unmarked version of your revised paper without tracked changes. You should upload this as a separate file labeled 'Manuscript'.

If you would like to make changes to your financial disclosure, please include your updated statement in your cover letter. Guidelines for resubmitting your figure files are available below the reviewer comments at the end of this letter.

If applicable, we recommend that you deposit your laboratory protocols in protocols.io to enhance the reproducibility of your results. Protocols.io assigns your protocol its own identifier (DOI) so that it can be cited independently in the future. For instructions see: https://journals.plos.org/plosone/s/submission-guidelines#loc-laboratory-protocols. Additionally, PLOS One offers an option for publishing peer-reviewed Lab Protocol articles, which describe protocols hosted on protocols.io. Read more information on sharing protocols at https://plos.org/protocols?utm_medium=editorial-email&utm_source=authorletters&utm_campaign=protocols.

As the corresponding author, your ORCID iD is verified in the submission system and will appear in the published article. PLOS supports the use of ORCID, and we encourage all coauthors to register for an ORCID iD and use it as well. Please encourage your coauthors to verify their ORCID iD within the submission system before final acceptance, as unverified ORCID iDs will not appear in the published article. *Only*  the individual author can complete the verification step; PLOS staff *cannot*  verify ORCID iDs on behalf of authors.

We look forward to receiving your revised manuscript.

Kind regards,

Rachel Suet Kay Chan, Ph.D.

Academic Editor

PLOS One

Journal Requirements:

If the reviewer comments include a recommendation to cite specific previously published works, please review and evaluate these publications to determine whether they are relevant and should be cited. There is no requirement to cite these works unless the editor has indicated otherwise.

Please review your reference list to ensure that it is complete and correct. If you have cited papers that have been retracted, please include the rationale for doing so in the manuscript text, or remove these references and replace them with relevant current references. Any changes to the reference list should be mentioned in the rebuttal letter that accompanies your revised manuscript. If you need to cite a retracted article, indicate the article’s retracted status in the References list and also include a citation and full reference for the retraction notice.

**Additional Editor Comments:**

[Note: HTML markup is below. Please do not edit.]

Reviewers' comments:

Reviewer's Responses to Questions

**Comments to the Author**

Reviewer #2: All comments have been addressed

**********

2. Is the manuscript technically sound, and do the data support the conclusions?

Reviewer #2: Yes

**********

3. Has the statistical analysis been performed appropriately and rigorously?

Reviewer #2: Yes

**********

4. Have the authors made all data underlying the findings in their manuscript fully available?

The PLOS Data policy

Reviewer #2: Yes

**********

5. Is the manuscript presented in an intelligible fashion and written in standard English?

Reviewer #2: Yes

**********

Reviewer #2: The authors have made substantial revisions in response to the previous round of review and the manuscript is considerably improved. In particular, the removal of inferential statistical testing (Fisher's exact tests, p-values) from Tables 2–4 and the abstract directly and adequately addresses the major concern raised regarding the skip-logic questionnaire design — the quantitative results are now presented appropriately as descriptive frequencies. The expanded justification of the Bourdieusian theoretical framework, the added methodological detail on the qualitative coding process (n=13, independent double-coding with consensus resolution), and the more cautious framing of "community trust" as researchers' perception rather than direct evidence are all welcome and responsive changes.

A few remaining issues should be addressed before publication:

1. A residual artifact of the skip-logic design remains in the prose, even though it was corrected in the tables. The Discussion states: "The statistical evidence is compelling: 100% of researchers who identified misinformation, mistrust, or language barriers also perceived a general challenge in the consent process." Given the questionnaire's branching logic, participants were only asked about specific consent barriers if they had already indicated a general consent difficulty — so 100% overlap is a structural property of the instrument, not an empirical finding. I would ask the authors to either remove this framing or explicitly acknowledge it reflects the skip-logic design rather than an independent result.

2. Claims are still generalized beyond the study setting in places that were not addressed in the previous revision. The Introduction and Discussion make several statements extending the findings to "LMICs," "the Global South," and "developing health systems" broadly (e.g., describing Fiocruz's experience as "a replicable model for other middle-income nations"). The authors have appropriately added hedging language regarding the sample size and representativeness elsewhere in the manuscript, but this same caution has not been extended to these broader geopolitical claims, which remain based solely on a convenience sample from six Fiocruz-funded projects in Brazil. I'd recommend qualifying these statements or explicitly noting them as speculative extrapolation.

3. The external comparator used to support the "academic whiteness" argument is not well matched to this study. The Discussion cites data from a different institution (UFMT) on racial composition of principal investigators to support claims of structural racial asymmetry within this study's sample (which measured all research team members, not just PIs). I would ask the authors to either better justify the comparability of these two data sources or soften the claim.

4. Out of the 67 participants recruited, 51 comprised the final sample; a brief breakdown of the 16 exclusions would improve transparency, though this is not essential.

**********

what does this mean?). If published, this will include your full peer review and any attached files.

If you choose “no”, your identity will remain anonymous but your review may still be made public.

**Do you want your identity to be public for this peer review?**  For information about this choice, including consent withdrawal, please see our Privacy Policy

Reviewer #2: **Yes:** Keshav Banga

**********

[NOTE: If reviewer comments were submitted as an attachment file, they will be attached to this email and accessible via the submission site. Please log into your account, locate the manuscript record, and check for the action link "View Attachments". If this link does not appear, there are no attachment files.]

To ensure your figures meet our technical requirements, please review our figure guidelines: https://journals.plos.org/plosone/s/figures

You may also use PLOS’s free figure tool, NAAS, to help you prepare publication quality figures: https://journals.plos.org/plosone/s/figures#loc-tools-for-figure-preparation.

NAAS will assess whether your figures meet our technical requirements by comparing each figure against our figure specifications.

# Author comment: Author response to Decision Letter 2

**DOI:** 10.1371/journal.pone.0359217.r004

7 Sep 2026

Dear Dr. Rachel Suet Kay Chan and Reviewers,

We thank the Academic Editor and Reviewer #2 for their thorough evaluation of our manuscript and for acknowledging the substantial improvements made during the previous revision. We value the constructive feedback, which enabled us to further enhance the accuracy, reflexivity, and transparency of our work.

Below, we present a point-by-point response detailing how each comment was addressed in the revised manuscript. All corresponding revisions have been highlighted in the "Revised Manuscript with Tracked Changes" file and incorporated into the "Manuscript" file (clean version).

Responses to Reviewer #2

Comment 1: A residual artifact of the skip-logic design remains in the prose, even though it was corrected in the tables. The Discussion states: "The statistical evidence is compelling: 100% of researchers who identified misinformation, mistrust, or language barriers also perceived a general challenge in the consent process." Given the questionnaire's branching logic, participants were only asked about specific consent barriers if they had already indicated a general consent difficulty — so 100% overlap is a structural property of the instrument, not an empirical finding. I would ask the authors to either remove this framing or explicitly acknowledge it reflects the skip-logic design rather than an independent result.

Response: We thank the reviewer for highlighting this oversight. We recognize that this 100% overlap was an artifact of the questionnaire's branching logic rather than an independent empirical finding. Accordingly, we have removed this inappropriate statistical framing from the Discussion. The revised text now presents these findings descriptively within the subgroup of participants who encountered difficulties during the consent process, without making unwarranted inferential claims.

• Location in text: Discussion [Page 25, Lines 556-560]): “…Among researchers who reported difficulties in the consent process, barriers such as participants' comprehension challenges, misinformation, and mistrust were particularly prominent.”

Comment 2: Claims are still generalized beyond the study setting in places that were not addressed in the previous revision. The Introduction and Discussion make several statements extending the findings to "LMICs," "the Global South," and "developing health systems" broadly (e.g., describing Fiocruz's experience as "a replicable model for other middle-income nations"). The authors have appropriately added hedging language regarding the sample size and representativeness elsewhere in the manuscript, but this same caution has not been extended to these broader geopolitical claims, which remain based solely on a convenience sample from six Fiocruz-funded projects in Brazil. I'd recommend qualifying these statements or explicitly noting them as speculative extrapolation.

Response: We thank the reviewer for this critical observation. We fully agree that drawing broad, systemic conclusions regarding "low- and middle-income countries" (LMICs), the "Global South," or "developing health systems" based on an exploratory convenience sample (n = 51)—comprising six research projects funded by Fiocruz—goes beyond the study's empirical basis. In response, we have revised the Introduction, Discussion, and Conclusion to eliminate sweeping generalizations, remove prescriptive statements regarding transferability, and explicitly qualify comparisons across different contexts as situated, exploratory reflections. Below, we present the key revisions made to align the text with the study's scope:

• Location in text: Introduction [Page 3, Lines 73-78]): “Given this extensive reach and expertise, Fiocruz’s institutional experience across a geographically heterogeneous territory provides valuable contextual insights that can inform broader discussions on clinical research governance in resource-limited settings, while acknowledging that direct transferability is inherently constrained by local systemic specificities.”

• Location in text: Introduction [Page 5, Lines 101-103]): “These obstacles, while observed within specific Brazilian research settings, illustrate operational and logistical challenges that may resonate across developing health systems...”

• Location in text: Introduction [Page 5, Lines 112-115]): “By addressing this need, this study provides a situated diagnosis of this institutional setting and offers an exploratory contribution to debates surrounding the operational sustainability of clinical research in resource-limited settings.”

• Location in text: Conclusion [Page 29, Lines 671-673]): “This multiplicity of perspectives can strengthen the design of clinical research, favoring approaches that are more sensitive and responsive to the operational and social realities of vulnerable territories in Brazil and potentially comparable settings.”

Comment 3: The external comparator used to support the “academic whiteness” argument is not well matched to this study. The Discussion cites data from a different institution (UFMT) on racial composition of principal investigators to support claims of structural racial asymmetry within this study's sample (which measured all research team members, not just PIs). I would ask the authors to either better justify the comparability of these two data sources or soften the claim.

Response: We thank the reviewer for this critical observation. We agree that our sample—which includes team members occupying diverse technical, operational, and research roles—cannot be directly equated to data restricted exclusively to principal investigators (project coordinators) at UFMT.

In response, we have:

1. Retitled the subsection to a more objective and analytically precise heading: “The geopolitics of scientific capital and demographic asymmetries”;

2. Softened the claim by explicitly noting that our sample comprised diverse operational and research roles rather than solely PIs;

3. Clarified that the UFMT institutional data are introduced solely as a broader contextual reference illustrating leadership disparity in the national academic field, rather than as a direct methodological counterpart;

4. Emphasized that direct equivalencies must be interpreted with caution.

• Location in text: Discussion [Page 20, Lines 442-443]): “The geopolitics of scientific capital and demographic asymmetries.”

• Location in text: Discussion [Page 21, Lines 457-461]): “While our sample encompassed research team members across diverse operational and technical roles rather than exclusively principal investigators, the demographic profile observed (68.6% white) resonates with broader structural asymmetries documented in the Brazilian scientific field. For contextual comparison, institutional data on research project leadership…”

• Location in text: Discussion [Page 21, Lines 464-466]): “Although direct equivalencies between general field team composition and principal investigator demographics must be drawn with caution, both indicators collectively illustrate...”

• Location in text: Discussion [Page 21, Lines 468 - 471]): “The persistence of these geographic and racial concentrations may hinder the incorporation of perspectives aimed at methodological and theoretical diversification, which are essential for fostering plural and inclusive knowledge in public health.”

Comment 4: Out of the 67 participants recruited, 51 comprised the final sample; a brief breakdown of the 16 exclusions would improve transparency, though this is not essential.

Response: We appreciate this suggestion and have incorporated the complete breakdown of the 16 exclusions into the Results section under "Participant profile" to enhance transparency and reporting standards.

Location in text: Results [Page 11, Lines 250 - 253]): “The 16 exclusions comprised: participants who signed the ICF but did not complete the questionnaire (n = 7), duplicate questionnaire entries (n = 3), and failure to meet the eligibility criterion of having at least one year of clinical field research experience (n = 6).”

We believe the manuscript has been greatly improved by incorporating these revisions. We are deeply grateful to the editors and reviewers for their guidance.

We look forward to hearing from you regarding the status of our submission.

Sincerely,

Renata Caldeira Diniz

On behalf of all co-authors

Attachment Submitted filename: Response_to_Reviewers_PONE-D-26-21934.pdf

# Peer review: Decision Letter 2

**DOI:** 10.1371/journal.pone.0359217.r005

**Authors:** Rachel Suet Kay Chan

10 Sep 2026

Challenges and opportunities in clinical field research: A Bourdieusian analysis of Fiocruz’s experience in Brazil

PONE-D-26-21934R2

Dear Dr. Diniz,

We’re pleased to inform you that your manuscript has been judged scientifically suitable for publication and will be formally accepted for publication once it meets all outstanding technical requirements.

Within one week, you’ll receive an e-mail detailing the required amendments. When these have been addressed, you’ll receive a formal acceptance letter and your manuscript will be scheduled for publication.

An invoice will be generated when your article is formally accepted. Please note, if your institution has a publishing partnership with PLOS and your article meets the relevant criteria, all or part of your publication costs will be covered. Please make sure your user information is up-to-date by logging into Editorial Manager at Editorial Manager® and clicking the ‘Update My Information' link at the top of the page. For questions related to billing, please contact billing support.

If your institution or institutions have a press office, please notify them about your upcoming paper to help maximize its impact. If they’ll be preparing press materials, please inform our press team as soon as possible -- no later than 48 hours after receiving the formal acceptance. Your manuscript will remain under strict press embargo until 2 pm Eastern Time on the date of publication. For more information, please contact onepress@plos.org.

Kind regards,

Rachel Suet Kay Chan, Ph.D.

Academic Editor

PLOS One

Additional Editor Comments (optional):

Reviewers' comments:

Reviewer's Responses to Questions

**Comments to the Author**

Reviewer #2: All comments have been addressed

**********

2. Is the manuscript technically sound, and do the data support the conclusions?

Reviewer #2: Yes

**********

3. Has the statistical analysis been performed appropriately and rigorously?

Reviewer #2: Yes

**********

4. Have the authors made all data underlying the findings in their manuscript fully available?

The PLOS Data policy

Reviewer #2: Yes

**********

5. Is the manuscript presented in an intelligible fashion and written in standard English?

Reviewer #2: Yes

**********

Reviewer #2: (No Response)

**********

what does this mean?). If published, this will include your full peer review and any attached files.

If you choose “no”, your identity will remain anonymous but your review may still be made public.

**Do you want your identity to be public for this peer review?**  For information about this choice, including consent withdrawal, please see our Privacy Policy

Reviewer #2: **Yes:** Keshav Banga

**********

# Editor report: Acceptance letter

**DOI:** 10.1371/journal.pone.0359217.r006

**Authors:** Rachel Suet Kay Chan

PONE-D-26-21934R2

PLOS One

Dear Dr. Diniz,

I'm pleased to inform you that your manuscript has been deemed suitable for publication in PLOS One. Congratulations! Your manuscript is now being handed over to our production team.

At this stage, our production department will prepare your paper for publication. This includes ensuring the following:

* All references, tables, and figures are properly cited

* All relevant supporting information is included in the manuscript submission,

* There are no issues that prevent the paper from being properly typeset

You will receive further instructions from the production team, including instructions on how to review your proof when it is ready. Please keep in mind that we are working through a large volume of accepted articles, so please give us a few days to review your paper and let you know the next and final steps.

Lastly, if your institution or institutions have a press office, please let them know about your upcoming paper now to help maximize its impact. If they'll be preparing press materials, please inform our press team within the next 48 hours. Your manuscript will remain under strict press embargo until 2 pm Eastern Time on the date of publication. For more information, please contact onepress@plos.org.

You will receive an invoice from PLOS for your publication fee after your manuscript has reached the completed accept phase. If you receive an email requesting payment before acceptance or for any other service, this may be a phishing scheme. Learn how to identify phishing emails and protect your accounts at https://explore.plos.org/phishing.

If we can help with anything else, please email us at customercare@plos.org.

Thank you for submitting your work to PLOS One and supporting open access.

Kind regards,

PLOS One Editorial Office Staff

on behalf of

Dr. Rachel Suet Kay Chan

Academic Editor

PLOS One
